# On good encodings for quantum annealer and digital optimization solvers

**DOI:** 10.1038/s41598-023-32232-0

**Published:** 2023-04-06

**Authors:** Alberto Ceselli, Marco Premoli

**Affiliations:** Department of Computer Science, Università degli Studi di Milano, 18, via Celoria, 20133 Milano, Italy

**Keywords:** Mathematics and computing, Computer science

## Abstract

Several optimization solvers inspired by quantum annealing have been recently developed, either running on actual quantum hardware or simulating it on traditional digital computers. Industry and academics look at their potential in solving hard combinatorial optimization problems. Formally, they provide heuristic solutions for Ising models, which are equivalent to quadratic unconstrained binary optimization (QUBO). Constraints on solutions feasibility need to be properly encoded. We experiment on different ways of performing such an encoding. As benchmark we consider the cardinality constrained quadratic knapsack problem (CQKP), a minimal extension of QUBO with one inequality and one equality constraint. We consider different strategies of constraints penalization and variables encoding. We compare three QUBO solvers: quantum annealing on quantum hardware (D-Wave Advantage), probabilistic algorithms on digital hardware and mathematical programming solvers. We analyze their QUBO resolution quality and time, and the persistence values extracted in the quantum annealing sampling process. Our results show that a linear penalization of CQKP inequality improves current best practice. Furthermore, using such a linear penalization, persistence values produced by quantum hardware in a generic way allow to match a specific CQKP metric from literature. They are therefore suitable for general purpose variable fixing in *core* algorithms for combinatorial optimization.

## Introduction

Dedicated hardware performing quantum annealing (QA) is currently available and steadily improving^1,2^. QA was independently proposed and refined multiple times^3–5^ and is based on the principles of adiabatic quantum computation^6–8^. The agreed present formulation of QA was introduced by Kadowaki and Nishimori^9^. QA is an alternative to the digital algorithm of simulated annealing^10^. The use of QA machines as *general purpose optimizers* to solve hard combinatorial optimization problems is in fact keep on gaining interests^11^.

The industrial state-of-the-art in QA machines is considered to be D-Wave^12^: their device is composed by a set of physical qubits connected by physical links. Not all pairs of qubits are directly connected: a topology is given, which can be formally described as a graph $G=(V,E)$, having one vertex $v \in V$ for each qubit, and one edge $e \in E$ for each physical link. The device is able to solve instances of the following Ising Spin Glass Ground State Problem (IM)^13^ in a probabilistic way:

IM $$\begin{aligned} \min ~H(s) =&\sum _{i,j \in E} J_{i,j} s_i s_j + \sum _{i\in V} b_i s_i \quad \quad \quad\\&s_i \in \{-1, +1\}&\forall i \in V \end{aligned}$$

An instance of (IM) is ‘programmed’ on the machine by setting biases $b_i$ for each spin $i \in V$ (*i.e.*, the magnetic field at site *i*) and coupling $J_{i,j}$ for each edge $(i,j)\in E$ (*i.e.*, the coupling strength between spins *i* and *j*).

As described in the literature^7^, the resolution of (IM) is carried out on a quantum processing unit (QPU) by adding a time-dependent quantum tunneling Hamiltonian to that of *H*(*s*). A corresponding Schrödinger equation is solved for such a time-dependent Hamiltonian: its resolution process approximates the tunneling dynamics of the system between different eigenstates of *H*(*s*).

The (IM) is NP-Hard: Ising models are equivalent to quadratic *unconstrained* binary optimization (QUBO), which considers quadratic functions to be minimized, with variable values restricted in the $\{0,1\}$ domain. In principle, the transformation among the two models is straightforward: a spin $s_i\in \{-1, +1\}$ and a binary variable $x_i\in \{0,1\}$ are related with the simple equations $s_i = 2x_i - 1$ and $x_i = \frac{s_i+1}{2}$.

**Figure 1:**
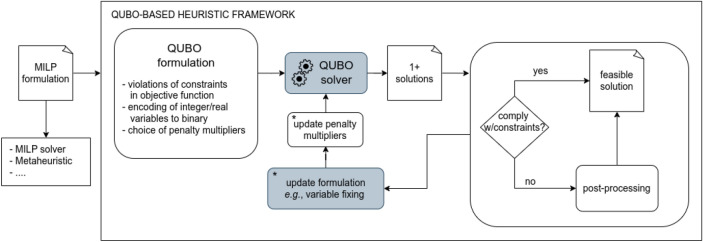
Schema of the QUBO-based heuristic framework to solve constrained combinatorial optimization problems.

A variety of *constrained* combinatorial optimization problems can currently be solved through QUBO relaxations on digital machines by traditional methods, which are extensively studied (and engineered) in the operations research community^14–17^. The process of solving constrained combinatorial optimization problems through quantum annealing, instead, is still a matter of research. It requires several transformation steps, summarized in the schema of Fig. 1.

First, the problem transformation into a QUBO requires modelling choices affecting the quality of solution. All constraints are *relaxed*; that is, they are replaced with a penalty term in the objective function representing the amount of their violation. Multipliers for the penalties must be set. Second, continuous and integer variables have to be encoded as binary variables. Third, when quantum annealing such as that of the D-Wave machine are considered, the interaction graph of the variables of the QUBO must match the graph of the machine. This mapping is obtained by the so-called minor-embedding^18–20^, which by itself is a NP-Hard problem^21^. The machine may yield different results for the same problem for distinct mappings.

Indeed, while *in principle* the advantage of quantum annealing over software running on digital machines is huge, no recent investigation proves *in practice* the former to produce better computational results than the latter, unless embedded as subroutine in problem-specific algorithms.

One reason is arguably constraints feasibility: there is no guarantee that solutions produced by solving the (unconstrained) QUBO satisfy all the constraints of the original combinatorial optimization problem. Compliance has to be checked after each execution, and restored by further processing if needed.

To the best of our knowledge, the only approach which proved to be able to directly exploit the characteristic of quantum annealing is the so-called *sample persistence*^22,23^: first introduced for simulated annealing^24^, it identifies variables whose value is persistent throughout repeated independent cycles of annealing. Such variables are candidate to be fixed to their persistent value^25^. The remaining ones are said to form a *core* of difficult variables, which then becomes the subject of search intensification^26^.

In this work we benchmark the performance of the bare QPU of D-Wave Advantage QPU^12^ in solving QUBO formulations derived from constrained combinatorial optimization problems. Our main focus is not on the design of a specific algorithm, but rather on identifying which techniques are more suited to improve those computational methods whose structure matches the pattern of Fig. 1. As a case study, we choose the cardinality constrained quadratic knapsack problem (CQKP), which extends QUBO in a minimal way, including only one inequality and one equality constraint. We experiment on four reformulations of CQKP as QUBO. We restrict our experiments to instances of CQKP whose interaction graph can be embedded in a D-Wave QPU machine.

We propose a comparison on two steps of the framework depicted in Fig. 1 (grey boxes in the figure). First, we assess computational results in solving the QUBO reformulation of CQKP, in terms of execution time and quality of solution. The goal is to test the advantage given by D-Wave as a general purpose solver for QUBO formulations derived from constrained combinatorial optimization problems. We compare the performance of D-Wave with two traditional approaches: the state-of-the-art general purpose solver based on mathematical programming Gurobi 9.5^27^ and the simulated annealing algorithm (SA)^10^, using its open-source CPU implementation provided by D-Wave^28^.

Second, we evaluate the goodness of values extracted via sample persistence from the solutions provided by D-Wave. The goal is to test the advantage to use D-Wave as a general purpose method to extract useful information from the solutions of the QUBO to solve the original CQKP. We compare these information with those provided by SA and by an ad-hoc measure on CQKP.

The paper is structured as follows. First, we present related works. In the following sections, we describe the mathematical models of CQKP and its four reformulations as QUBO, and we present experimental set-up and results. In the last section conclusions are drawn.

## Related works

### Benchmarking

Several benchmarking works on D-Wave quantum annealing processing unit are present in the literature.

Jünger et al. provide a seminal work of benchmarking^21^. They provide an experimental comparison for the resolution of the max-cut problem of D-Wave 2000Q (the version of the QPU with 2048 qubits preceding Advantage), a branch-and-cut and semidefinite programming algorithms and an ad-hoc heuristic tailored on the grapth of the QPU. For the sake of fairness, they consider only instances that fits the graph of the quantum hardware, the so-called ‘Chimera’ graph, *i.e.*, that do not need embedding. In their experiments D-Wave was able to provide, in a black-box set-up, solutions very near to the ground state energy ($<{}1\%$ of optimality gap); however, a traditional heuristic tailored on the Chimera graph performed better than D-Wave both in terms of quality and execution time. Their work was not replicated on Advantage machine, with 5640 qubits, and does not consider constrained problems requiring embedding to be solved by D-Wave.

Oshiyama and Ohzeki compare the performance on several classes of problem of four QUBO solvers^29^: the D-Wave Hybrid Solver Service, the Toshiba Simulated Bifurcation Machine, the Fujitsu Digital Annealer and a traditional simulated annealing algorithm. Experiments showed mixed outcomes, with no clear winner between the three quantum-inspired solvers, which had sometimes worst performances than the traditional simulated annealing algorithm. Moreover, they do not consider the performances of the bare QPU of D-Wave, but rather a resolution service provided by D-Wave hybridizing the QPU with traditional approaches whose details are not known to the end-user.

Several works focus on comparing different version of D-Wave QPU, or different quantum-inspired solvers, without considering traditional solver. Willsch et al. compare D-wave 2000Q with its successor D-Wave Advantage on the exact cover problem^30^. Their tests consider increasing instance sizes and increasing density of nodes connections. While Advantage outperforms 2000Q on largest problem, both in terms of solution quality and execution time, 2000Q provides better solution on problems with sparse connections and small size. Huang et al. compare D-wave Advantage and Fujitsu digital annealer on three combinatorial optimization problems^31^. Authors experiment on the decomposition of instances with size greater than the hardware limits, providing a schema based on block-structure of the problem formulation. In their conclusions, authors state that while the annealers provide good solutions on instances of small size and sparse connectivity, they struggle when facing large size or dense instances, and hence a decomposition algorithm is needed to improve solution quality and to reach feasibility.

Codognet presents a preliminary performance assessment of D-Wave Advantage and D-Wave hybrid software for the resolution of constrained optimization problems^32^, concluding that current generation of quantum annealers are not yet able to deal with constrained problems and must be embedded in a hybrid algorithm.

### QUBO formulations

Lucas provides a seminal work for the design of optimization algorithms inspired by adiabatic quantum^33^, presenting Ising formulation for many NP-hard problems. Glover et al. presents a didactic survey on QUBO formulation of combinatorial problems^34^.

Theoretical background to transform fully connected interactions of quadratic terms into linear terms is present in literature^35,36^. The resulting reformulation has a traditional counterpart in the Lagrangian relaxation of constraints. This latter needs the search for the best Lagrangian multipliers, and hence it needs an iterative process of sampling for the update of such multipliers. Kuramata et al. experiment this Lagrangian formulation on the quadratic assignment problem with D-Wave Advantage^37^. Authors were not able to find a fair amount of feasible solutions with the sole QPU of D-Wave, and had to resort to a post-processing procedure to restore feasibility. None of these last two works include standard techniques to update Lagrangian multipliers, which still need to be investigated.

Comparison between encodings of integer variables to binary variables has been investigated^38,39^. Authors compare binary, unary and one-hot encodings on the quadratic knapsack problem using Fujitsu Digital Annealer. Authors experiment with increasing instance sizes and take into consideration the rate of feasible solutions found, finding unary encoding to provide the best results.

## Cardinality constrained quadratic knapsack problem

The cardinality constrained quadratic knapsack problem (CQKP) is the problem of selecting exactly *k* items from a set, maximizing a profit defined by a quadratic function, and such that the weight of the selected items does not exceed a threshold.

Let *I* be the set of items to select and $x_i\in \{0,1\}$ be binary variables for each item $i\in I$, with value 1 if element *i* is selected, 0 otherwise. The formulation of CQKP is the following:

1 $$\begin{aligned} \max ~&\sum _{i \in I} l_i x_i + \sum _{(i,j) \in I\times I:j > i} q_{i,j} x_i x_j&\end{aligned}$$

CAP $$\begin{aligned} \text {s.t.~} \sum _{i \in I} a_i x_i \le b \end{aligned}$$

CARD $$\begin{aligned} &\sum _{i \in I} x_i = k\\&x_i \in \{0,1\} \end{aligned}$$

Constraint (CAP) imposes a limit on total ‘weight’ of selected items with $a_i \in \mathbb {R}_{\ge 0}$ being the weight of item $i\in I$ and $b \in \mathbb {R}_{\ge 0}$ being the limit on total weight of the selected items. Constraint (CARD) imposes to select exactly $k \in \mathbb {Z}_{\ge 0}$ items. Objective function (1) maximizes the ‘profit’ of selected items and is composed by a linear and a quadratic component: $l_i \in \mathbb {R}_{\ge 0}$ is the profit given by selecting item $i\in I$; $q_{i,j}\in \mathbb {R}_{\ge 0}$ is the profit given by selecting items *i* and *j* together, where matrix *q* is symmetric.

### QUBO formulation of CQKP

CQKP is formulated as a QUBO by relaxing both constraints (CAP) and (CARD), adding a penalty term in the objective function involving the amount of their violation. There are two standard reformulation approaches: (i) considering the sum of the square of the violations of each constraint formulated as an equality; in this case the penalties are an additional QUBO term in the objective function; (ii) considering the sum of linear violations of each constraint in the objective function, in a traditional Lagrangian relaxation approach^40^. In both cases, violations are multiplied by additional penalty values, whose optimal value should be found in order to obtain the best relaxed solution.

#### Square of violations of equality constraints

The relaxation of the equality constraint (CARD) is formulated as a QUBO as:

$$\begin{aligned} H^{\textsc {qubo}{}}_{\text{(CARD)}} = \left( \sum _{i \in I} x_i - k \right) ^2 \end{aligned}$$

Inequality constraint (CAP) is first formulated as an equality with the introduction of the slack variable $s \in \mathbb {Z}_{\ge 0}$:

2 $$\begin{aligned} \sum _{i \in I} a_i x_i + s = b \end{aligned}$$

This additional variable must be encoded as a set of binary variables to comply with the QUBO format; the choice of encoding results in different formulations. We experiment on binary and unary encoding for the slack variable.

With the unary encoding, variable *s* is replaced by *b* binary variables with unary coefficients. The resulting QUBO is:

$$\begin{aligned} H^{\textsc {qubo}{}, \textsc {unary}{}}_{\text{(CAP)}} = \left( \sum _{i \in I} a_i x_i + \sum _{j \in [1,b]} s_j - b \right) ^2 \end{aligned}$$

Unary coefficients result in lower value of quadratic coefficients; however, unary encodings requires a high number of additional variables and a highly degenerate formulation (*i.e.*, a value *v* for the original variable *s* is encoded by $$\left( {\begin{array}{c}b\\ v\end{array}}\right)$$ combinations of variables $s_j$ to value 1).

With the binary encodings, variable *s* is replaced by $M = \lfloor \log _2 \left( \sum _{i \in I} a_i - b \right) \rfloor + 1$ binary variables, where variable $s_m, \forall m \in [0,M]$, is multiplied by a coefficient $c_m$ defined as:

$$\begin{aligned}&c_m = {\left\{ \begin{array}{ll} 2^m &{} \text {if } m \in [0, M-1] \\ \left( \sum _{i \in I} a_i - b\right) + 1 - 2^M &{} \text {if } m = M \end{array}\right. }&\forall m \in [0, M] \end{aligned}$$

The resulting QUBO is:

$$\begin{aligned} H^{\textsc {qubo}{}, \textsc {binary}{}}_{\text{(CAP)}} = \left( \sum _{i \in I} a_i x_i + \sum _{j \in M} c_j s_j - b \right) ^2 \end{aligned}$$

Binary encoding requires a limited number of additional variables; however, the use of coefficients $c_m$ results in high quadratic coefficients, hence requiring a careful choice of the penalty multiplier for the QUBO, and with risk of numerical issues.

We do not take into consideration one-hot encoding. It combines the *cons* of unary and binary formulations: it leads to instances with high number of variables and high values of coefficients. Moreover, it also requires an additional constraint that must be relaxed.

#### Linear violations of equalities

As in traditional Lagrangian relaxation, constraints are relaxed by adding their violations to the objective function:

$$\begin{aligned}{} & {} H^{\textsc {linear}}_{\text{(CARD)}} = \sum _{i\in I} x_i - k\\{} & {} H^{\textsc {linear}{}}_{\text{(CAP)}} =\sum _{i \in I} a_i x_i - b \end{aligned}$$

#### QUBO formulation of CQKP

The QUBO formulation to minimize CQKP is:

3 $$\begin{aligned} H_{\text {CQKP}} = \sum _{i\in I} -l_ix_i + \sum _{(i,j) \in I\times I :j > i} - q_{i,j} x_i x_j + \lambda _{(CARD)} H_{\text{(CARD)}} + \lambda _{\text{(CAP)}} H_{\text{(CAP)}} \end{aligned}$$

where $\lambda _{\text{(CARD)}} \in \mathbb {R}$ and $\lambda_{\text{(CAP)}}\in \mathbb {R}$ are penalty multipliers for the violations of the corresponding constraints. We experiment on 4 formulations of (3), with 4 combinations of relaxation of the constraints.

- binary formulation, with both (CARD) and (CAP) relaxed as QUBO, with binary encoding of the slack variable of (CAP):
- unary formulation, with both (CARD) and (CAP) relaxed as QUBO, with unary encoding of the slack variable of (CAP).
- qubo-card formulation, with (CARD) relaxed as QUBO and (CAP) relaxed linearly.
- linear formulation, with both (CARD) and (CAP) relaxed linearly.

## Experiments

We experiment on instances from literature^41^, characterized by the number of items in set *I* in set $\{50, 70, 100\}$ and by the density of non-zeros values of quadratic profit coefficients in matrix *q* in set $\{25\%, 50\%, 75\%, 100\%\}$. Ten instances have been generated for each combination of density and number of items, for a total of 120 instances.

We chose to limit our experiments to instances of 100 items. While D-Wave Advantage has 5640 qubits, it can embed a clique with maximum 177 variables when all qubits and links of the machine are working^42^. However, each broken qubit or link decreases the size of the clique that can be embedded^43^. Moreover, performances decrease as the size of the clique increases. At the same time, traditional solvers struggle when the size of the problem reaches 100 items^41,44^.

For the unary formulations, we executed D-Wave on 41 instances out of the complete set of 120; the size of the instances not considered was too high to fit D-Wave machine.

### Setup of solvers

Following D-Wave rule-of-thumbs we set: ‘annealing time’ to $100\,\upmu s$; ‘anneal offset’ to $\alpha (\log 2^{(1-c)/c} - 1)$, where *c* is the length of the chain to which apply the offset and $\alpha$ is set to value 0.2; ‘chain strength’ to the value given by the *uniform torque compensation* function provided by D-Wave. This latter parameter is the penalty term multiplying violations of equalities of the chain of copies of a variable.

For binary and unary formulations a single embedding per instance have been computed. For qubo-card and linear formulations three embeddings have been computed, one for each number of items of the experimental instances.

Parameters of Gurobi are left to default values, with a time limit of 60 seconds for its execution. Gurobi stops its execution when it is able to prove optimality of its solution, or after reaching the time limit. Parameters of SA are left to default values.

As a further solving approach, we consider the draw at random of as many solutions as possible in a fixed amount of time, selecting the best solution found. In particular, we set the execution time to be equal to the execution time of D-Wave. To build a solution we pick *k* items. The probability to draw item $i\in I$ is computed starting from the following measure:

$$\begin{aligned} g_i = \frac{l_i+\sum _{j\in I} q_{i,j}}{a_i}, \forall i \in I \end{aligned}$$

to which we refer as ‘potential gain’. The probability of draw is given by the normalization of all potential gains:

4 $$\begin{aligned} p_i = \frac{g_i}{\sum _{i\in I} g_i} \end{aligned}$$

#### Key performance indicators

We take into consideration two types of Key Performance Indicators (KPI).

First, as common computational KPIs, we consider the total execution time and the value of the objective function yielded by each solver. Second, more specifically, we evaluate the usefulness of persistence of values in the set of solutions yielded by a solver. We introduce a KPI to evaluate how much the information extracted from a set of samples can be used for search intensification in general purpose resolution procedures. Let us consider (i) a sorting *S* of the variables *x* in the QUBO, (ii) the value $x'$ of such variables in a heuristic solution, and (iii) the value $x^\star$ of such variables in the best known solution. The intuition is that only a *small core* of binary decision variables often exists, which are difficult to set in the optimization problem, thus requiring implicit enumeration procedures, while the remaining ones are easy to fix. Therefore, *core* methods^45^ for combinatorial optimization consist in building *S* by putting each decision variable in order of *confidence* that its value in the optimal solution is actually the same value taken in $x'$. That is, a promising fixing is performed by choosing first those variables appearing at the beginning of *S*, and fixing their values to $x'$, allowing the search only for value of the remaining variables by a suitable optimization algorithm^26^. Let *w*(*S*) be the index of the first variable in the sorting *S* such that $x'_{w(S)} \not = x^\star _{w(S)}$. Intuitively, *w*(*S*) is the first position in which a core algorithm, fixing variables in the order of *S*, would make an error. We will refer to *w*(*S*) as ‘error point’.

Accordingly, let *w*(*R*) be the error point obtained by a uniform random sorting *R*. Let *Q* be the sorting obtained by using the persistence values of a sample of solver solutions. In the CQKP case, let $x'$ be defined by setting to 0 the $|I|-k$ variables with lowest persistent value (setting to 1 the remaining *k* variables). Let *w*(*Q*) be the corresponding error point. As KPI for the quality of a particular formulation we therefore consider the probability that the random sorting performs better than the sorting induced by the solver. We formally define such probability as:

5 $$\begin{aligned} { \Pi (Q) = {P[w(R) \ge w(Q)] \approx } \left( {\begin{array}{c}|I|-k\\ w(Q)\end{array}}\right) /\left( {\begin{array}{c}|I|\\ w(Q)\end{array}}\right) } \end{aligned}$$

The denominator counts in how many ways *w*(*Q*) variables can be chosen at random, while the numerator counts how many of these subsets are variables of value 0 in the CQKP optimal solution (which are those a core algorithm would correctly exclude from the search). Formally, the expression of $P[w(R) \ge w(Q)]$ is exact when $w(Q) \le |I| - k$, since in this case only variables whose optimal value is 0 are correctly fixed. According to the parameters of our test instances, when $w(Q) > |I| - k$, the probability $P[w(R) \ge w(Q)]$ becomes negligible (nevertheless, the complete exact formula and its interpretation are reported in the supplementary material). By using $\Pi (Q)$ to measure the quality of *Q* and $x'$ in a randomized method, instead of more common metrics for specific solutions^22,23^, we implicitly evaluate which impact various formulations can have in *any* core algorithm. The lower the $\Pi (Q)$ value, the better.

### Computing effort

**Figure 2:**
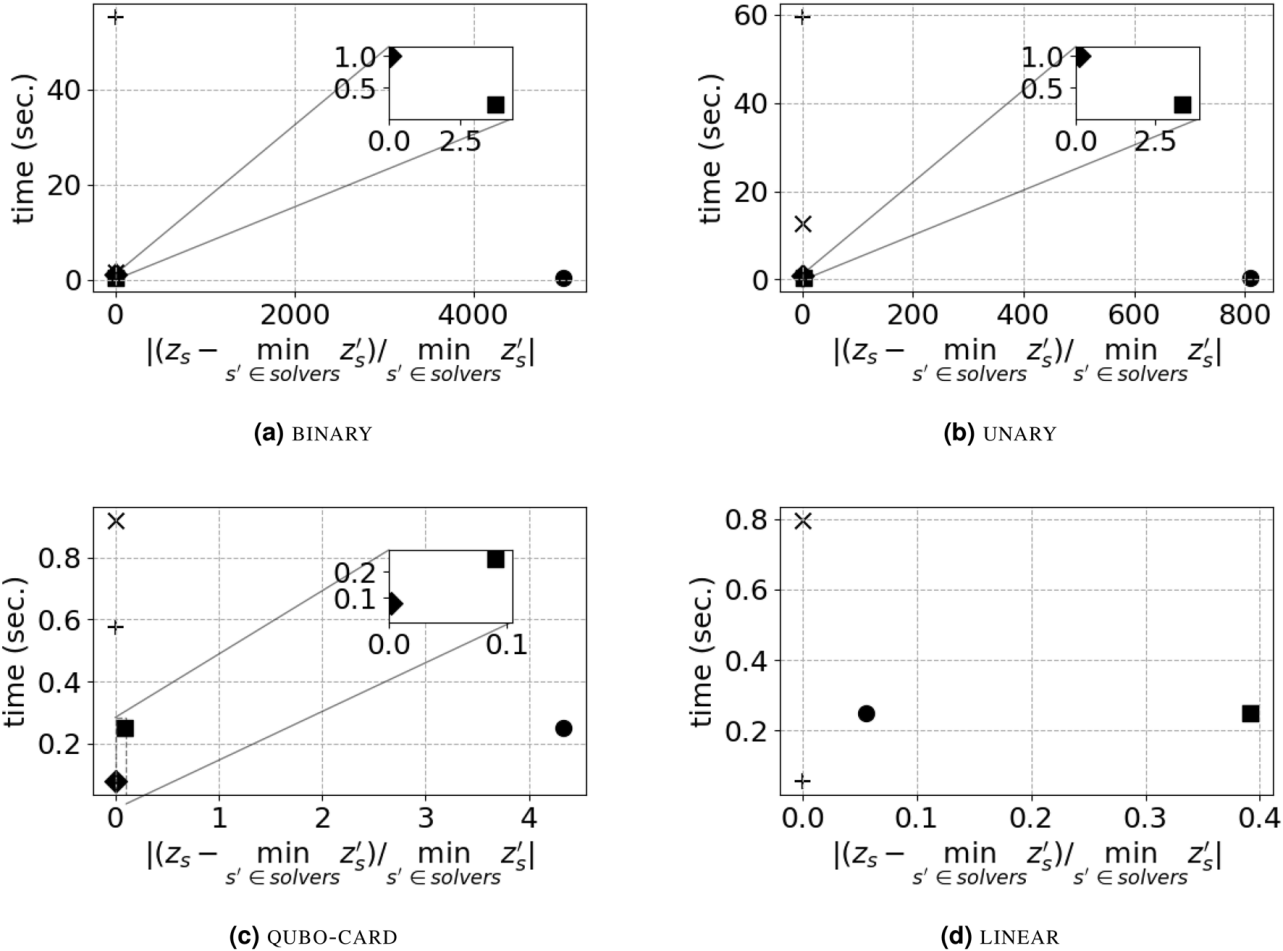
Scatter plots of execution time (on *y*-axis) and the relative difference between the objective function value $z_s$ resulting from a solver *s* and the minimum among all solvers (on *x*-axis), *i.e.*, $|\frac{z_s - \min _{s'\in \text {solvers}} z_{s'}}{ \min _{s'\in \text {solvers}} z_{s'}}|$ . Values are averaged on all instances.  randam + Gurobi  D-Wave × SA  Gurobi 1sec.

Figure 2 shows the comparison of computational results. Each sub-figures is related to a QUBO formulation: Fig. 2a for binary formulation, Fig. 2b for unary formulation, Fig. 2c for qubo-card formulation and finally Fig. 2d for linear formulation. Each sub-figure is a scatter-plot with one point per solver, whose coordinates are the execution time on *y*-axis, while the *x*-axis contains the relative difference between the value $z_s$ of the QUBO yielded by a solver *s* and the minimum value of the QUBO among all solvers, *i.e.*, $|(z_s - \min _{s'\in \text {solvers}} \{z_s'\})/ \min _{s\in \text {solvers}} \{z_s'\} |$. The closer a point is to the origin of the plane, the better. Figure 2a–c also show the magnification of the region of the plane containing points closer to the origin. Each value is averaged over all density values, all size of set of item *I* and all the 10 instances for each combination of density and number of items: *i.e.*, each value is the average over 120 results. We restrict to this case as: (i) we did not notice differences in results provided by different density of quadratic coefficient matrix *q*, and (ii) we noticed minor differences arising by different number of items *I*, that are described in Supplementary Materials. Values of coordinates of all points of Fig. 2 and the complete results are reported in the Supplementary Material, Tables S2.1– S2.6.

The computation time of D-Wave results from the set-up of the execution parameters; hence, it has fixed value that is not influenced by the size of the instance solved, as long as the instance fits the hardware graph. In the set-up of our experiments D-Wave execution is in the range [0.2, 0.3] seconds. Such computation time does not include the time to perform minor embedding. On average, embedding took $\sim 30$ seconds for instances with 50 variables, $\sim 100$ seconds for instances with 70 variables and $\sim 330$ seconds for instances with 100 variables. It is fair not to consider such time in the comparison, as a single embedding can be pre-computed and used for each instance with the same interaction graph. In our case, such interaction graph is always a clique.

We also take into consideration results of Gurobi stopped after 1 second of execution (labelled as ‘Gurobi 1sec’ in the figures) and of the best solution found by drawing at random as many solutions as possible in 0.25 seconds (labelled as ‘random’ in the figures). The rationale is to compare the goodness of solvers which are executed in the same magnitude of time as D-Wave.

Clear patterns arise in the figures: D-Wave solutions lie in the bottom-right of the plane. Traditional state-of-the-art mathematical programming solver Gurobi lies either in the top left or in bottom left of the plane. Traditional heuristic SA lies in the left part of the plot. Traditional fast methods of random draw and Gurobi stopped after 1 second lies closer to the origin than both D-Wave and Gurobi with 60 seconds time limit.

However, the value of the minimum solution found by D-Wave is never the lowest among all solvers. These findings match recent literature^21,32^.

The comparison of different formulations (*i.e.*, sub-figures in Fig. 2) allows to go one step beyond. Striking differences arise between formulations in which inequality constraint (CAP) is relaxed as QUBO (binary and unary), and formulations in which (CAP) is relaxed linearly (qubo-card and linear). For formulations binary and unary the solution provided by D-Wave is hundreds of times higher than the best solution found. For formulation qubo-card the solution provided by D-Wave is $\sim 4$ times higher than the best, while for formulation linear is close to the best ($\sim 5$% worse).

For binary and unary, Gurobi either reaches, or is close to, the time limit of 60 seconds of execution. We recall that in our setting Gurobi stops execution only when it is able to guarantee optimality of the solution found. For qubo-card formulation, where the equality constraint (CARD) is relaxed as QUBO, Gurobi is able to prove optimality of its solution in reasonable time ($\sim 0.6$ seconds), while D-Wave is not able to find good heuristic solutions.

SA is always able to find solutions close to the best: for binary and unary formulations the solutions of SA are $\sim 11\%$ and $\sim 15\%$ worse than the best, while for qubo-card and linear formulations the solutions of SA are either equal or very close to the best solutions. The execution time of SA is highly related to the number of variables: for binary, qubo-card and linear formulations SA takes $\sim 1.5$, $\sim 0.9$ and $\sim 0.8$ seconds, respectively; for unary formulation instances, which have on average 140 variables for $|I|=50$ case, 180 variables for $|I|=70$ case and 220 variables for $|I|=100$ case, SA takes $\sim 12$ seconds on average.

Only 1 second of execution of Gurobi suffices to find good solution ($\sim 3.5\%$ worse than best in binary case, $\sim 10\%$ worse than best in unary case, and the best solution in qubo-card and linear formulations).

Random solutions are better than those of D-Wave in binary, unary and qubo-card formulations, but still far from the best found (from 10% worse to 3 times worse than the best). In linear case, random solutions are worse than D-Wave solutions, and $\sim 40\%$ worse than the best solution found.

#### Discussion

binary and unary formulations have additional complexity given by the encoding of the integer slack variable of the inequality (CAP), which is required for its formulation as QUBO. As discussed in the previous section, encodings have different *cons* which lead to higher complexity: binary encoding increases values of quadratic coefficients, while unary encoding increases the number of variables to consider. The increased complexity results: (i) in a higher computation time for Gurobi, which is able to quickly find good solution while struggling in proving their quality; (ii) in a minor worsening of solution and higher computation time for SA; and (iii) in a major worsening of results for D-Wave, whose solutions are hundreds or thousands of times worse than the best solution found, and worse than random drawn of solutions in the same amount of time.

In qubo-card and linear formulations the quality of D-Wave solutions improves, but still D-Wave is outperformed by traditional solvers. Indeed, in linear case, when both constraints are relaxed linearly, Gurobi is able to find a proven optimal solution faster than the execution of D-Wave to find heuristic solution.

Summarizing, when each scatter-plot is analyzed independently, our results confirm those from the literature: in each formulation D-Wave solutions are dominated by at least one traditional solver. The fixed execution time provided by D-Wave, not linked to the size of the instance solved, is a promising feature, that may be useful if hardware of bigger size and improved accuracy becomes available.

Instead, when comparing scatter-plots with each other, our results are more insightful: using simple linear penalties lead to a decrease of orders of magnitude in the gap $|(z_s - \min _{s'\in \text {solvers}} \{z_s'\})/ \min _{s\in \text {solvers}} \{z_s'\} |$, in *x*-axis. Previous analyses^35–37^, exploiting more involved linearization techniques, focused on problem size reduction, without proposing comparison with different formulations. Our results show that a linear penalization approach is not only useful to overcome limits in problem size, but has an actual impact on the *quality* of the solutions produced by the D-Wave QPU.

### Sample persistence

**Figure 3:**
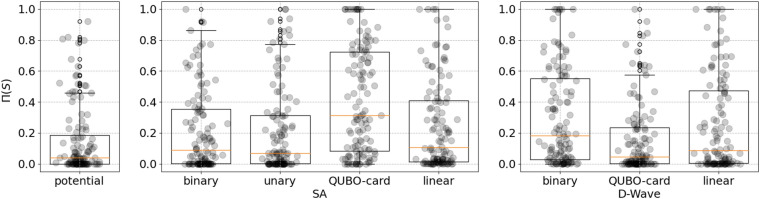
Comparison of $\Pi (S)$ values of different sorting methods. From left to right: the sorting by increasing value of the *potential gain* measure (4), the sorting by increasing average variable values in SA solutions over repeated starts, and the sorting by increasing average values in D-wave samples. Orange bars represent median values, which in details are 0.039 for the *potential gain* measure (4), 0.089 for SA-binary, 0.068 for SA-unary, 0.106 for SA-linear, 0.311 for SA-qubo-card, 0.182 for D-Wave-binary, 0.044 for D-Wave-qubo-card and 0.087 for D-Wave-linear.

Figure 3 shows boxplots of values of KPI $\Pi (S)$. The first boxplot contains value of KPI $\Pi (S)$ computed from the sorting provided by the ascending value of the ad-hoc ‘potential gain’ measure (4); the second and third block of boxplots contain results of the sorting computed as the ascending mean values of variables on the complete set of solutions created during the execution of SA and D-Wave, respectively. For SA we take into consideration results of all 4 formulations of testing, while for D-Wave we do not consider unary formulation, for which it has been possible to execute only 41 instances out of 120. Results of this latter scenario and tables with complete results are discussed in Supplementary Materials. Error points *w*(*S*), needed to calculate $\Pi (S)$ in (5), are computed using the integer feasible solution $x^\star$ given by solving the original instance of CQKP with Gurobi, with a time limit of 1 hour.

We recall that the lower value of $\Pi (S)$, the better. The sorting provided by the ad-hoc measure of potential gain yields the best value of $\Pi (S)$, with a median value of 0.033. SA median values are always worse: for qubo-card and linear cases, for which SA is able to find optimal solution of the relaxation, the median values are 0.311 and 0.106, respectively; for binary and unary, for which SA found a solution $\sim 10\%$ worse than the best found, the median value of the KPI is 0.089 and 0.068, respectively.

D-Wave has worse median values for binary and linear formulations, respectively 0.182 and 0.087. The median result for qubo-card case instead is 0.044, which is remarkably very close to the value of the potential gain measure.

#### Discussion

The introduction of our new KPI $\Pi (S)$ allows to highlight an insightful and unexpected result: D-wave in qubo-card formulation has comparable performance to the ad-hoc potential gain measure. Additionally, D-Wave is generic, unaware of the features of problem underlying the objective function it is solving, while potential gain measure is tailored on the CQKP problem. In this perspective, D-Wave might be used as a general purpose generator of a sorting, exploiting persistence to drive the selection of a *core* of decision variables which are difficult to set.

## Conclusion

In this work we compared performances of four QUBO formulations for the CQKP and three QUBO solvers, with a focus on the bare QPU of D-Wave Advantage. In our comparisons we considered two aspects: the pure computational results and the goodness of information retrieved by sample persistence.

From the computational point of view, while the comparisons among solvers in distinct formulations substantially match the literature, the observations coming from the comparison of different formulations provides interesting insights. The best performance provided by D-Wave was given by the formulation in which the inequality constraint of CQKP was relaxed linearly. This is not matching the current best practice, which instead advises to transform it in a quadratic fashion. We explain this phenomenon mainly as follows: the linear relaxation allows to avoid the explicit addition of the slack variable of the constraint, which in turns would require to be encoded as a set of binary variables. More in details, the QUBO resulting from linear penalties has much more sparse interaction graph. All existing QPU architectures are build with a limited number of direct connections between qubits. When more connections than physical ones are required, chains of interconnecting qubits are needed. The coherence of their values is known to be an issue, and the QPU of D-Wave Advantage struggles to find good solution when facing such highly connected QUBOs. Being the objective function of the model with linear penalties more sparse, its embedding in the QPU requires much less qubits in these chains, ultimately leading to better performance.

On the analysis of sample persistence, we introduced a new KPI to evaluate how much the information extracted from sample persistence can be used for search intensification in general purpose resolution procedures. The most insightful result was the following: D-Wave using a linear relaxation instead of the quadratic one carries persistence information that shows experimentally to be representative of an optimal solution. Specifically, it matches an ad-hoc metric on CQKP in identifying *cores* of variables which are difficult to set. Since the ad-hoc metric exploits specific combinatorial features of the CQKP, while D-Wave does not, the latter is applicable in a wider setting.

## Supplementary Information

Supplementary Information.

## Data Availability

Replication data are freely available at https://doi.org/10.13130/RD_UNIMI/Y3GKUF.
