# Supplementary material for: On good encodings for quantum annealer and digital optimization solvers
Source: Sci Rep. 2023 Apr 6;13:5628. doi: 10.1038/s41598-023-32232-0 (PMC10079660; doi:10.1038/s41598-023-32232-0)
Supplement: Supplementary file 1 — Supplementary Information. [file 41598_2023_32232_MOESM1_ESM.pdf]

‘On good encodings for quantum annealer and digital optimization solvers’ – Supplementary Materials

Alberto Ceselli[1] and Marco Premoli[1,*]

[1]Università degli Studi di Milano, Department of Computer Science, 18, via Celoria, 20133, Milano, Italy
[*]Corresponding author: marco.premoli@unimi.it

S1 Exact formula for the $\Pi(S)$ KPI

Hereafter we report the formal computation of the probability $P[w(R) \geq w(Q)]$, used in the paper for defining our KPI $\Pi(S)$.

$$\Pi(Q) = P[w(R) \geq w(Q)] = \begin{cases} \binom{|I|-k}{w(Q)} / \binom{|I|}{w(Q)} & \text{if } w(Q) \leq |I| - k \\ \binom{k}{k-l} / \binom{|I|}{|I|-k, l, k-l} & \text{otherwise} \end{cases} \quad (1)$$

When $w(Q) \leq |I| - k$, the denominator counts in how many ways $w(Q)$ variables can be chosen at random, while the numerator counts how many of these subsets are variables of value 0 in the CQKP optimal solution (which are those a core algorithm would correctly exclude from the search).

When $w(Q) > |I| - k$, $l = |I| - w(Q)$ represents the number of variables correctly fixed to 1 by Q. The intuition is the following: a random sorting needs to partition I in three subsets: those variables that are at 0, those variables that are at 1, and have been fixed to 1 also by Q, and the remaining ones (still among the k having value 1 in an optimal solution). The denominator counts the number of such partitions. Among all these partitions, there are as many correct ones as the possible subsets of l elements from the set of k variables having value 1 in an optimal solution (and so counts the numerator). The approximation of the second case to value 0 is justified by the parameters of the instances used in our tests. For example, assuming $|I| = 100$, $k = 24$, and $w(Q) = |I|$, the probability of random to perform at least as good as Q is about 10^{-22}.

S2 Computational results - extended tables

Table S2.2 contains the value of coordinates of points in all sub-figures of Figure 2 in the paper. Each sub-table refer to a formulation and the corresponding sub-figure of Figure 2: Table S2.2a for BINARY, Fig. 2a; Table S2.2b for UNARY, Fig. 2b; Table S2.2c for QUBO-CARD, Fig. 2c; Table S2.2d for LINEAR, Fig. 2d. Each row in a sub-table refers to a instance size in terms of number of items $|I| \in \{50, 70, 100\}$. The second column identifies the coordinate: time in seconds for the y-axis and the formula $|\frac{(z_s - \min_{s' \in solvers} z'_s)}{\min_{s' \in solvers} z'_s}|$ for the x-axis. The successive columns refer to the solvers.

Tables S2.3, S2.4, S2.5 and S2.6 contain computational results of BINARY, UNARY QUBO-CARD and LINEAR scenarios, respectively. The first block of each Table contains the properties of an instance, *i.e.*, density of the non-zero values of quadratic profit coefficients q, number of items and index of the instance. The successive blocks contains objective function value (‘obj. value’) and computational time in seconds (‘time’) for the solvers considered. methods considered. All tables have results of Gurobi, SA, D-Wave and the random drawn of solutions (labelled ‘random’); Tables S2.3, S2.4 and S2.5 also contains results of Gurobi stopped after 1 second of execution (labelled ‘Gurobi 1sec.’). The block of Gurobi for Tables S2.3 and S2.4 also contains the optimality gap obtained at the end of the execution (column ‘opt. gap’). Tables S2.5 and S2.6

do not contain such field, as the optimality gap is either zero (*i.e.*, the optimal solution have been found) or very close to zero. In Table S2.4, instances for which the execution was not possible for D-Wave solver are labelled with symbol '-'.

Execution time of Gurobi has a minimum resolution of 0.01 seconds; entries of value 0 have meaning < 0.01. Execution time of D-Wave is the value of parameter `qpu_access_time` returned by D-Wave API.[1]

Table S2.7 contains the results of KPI $\Pi(S)$. The first block of each Table contains the properties of an instance, *i.e.*, density of the non-zero values of quadratic profit coefficients q, number of items, index of the instance and the value of parameter k. The successive blocks refers to the sorting provided by the potential gain formula (9), by SA and by the sampling of D-Wave. These latter blocks contains results for the four formulations experimented. Each block contains the index w of the first variable in the sorting whose value differs from that of the best known solution and the corresponding value of KPI $\Pi(S)$ (computed with (8)). For the column of D-Wave in UNARY formulation, instances for which the execution was not possible are labelled with symbol '-'.

Fig. S2.1 shows the comparison of computational results. Each row of sub-figures is related to a QUBO formulation: Fig. S2.1a for BINARY formulation, Fig. S2.1b for UNARY formulation, Fig. S2.1c for QUBO-CARD formulation and finally Fig. S2.1d for LINEAR formulation. Each column of sub-figures contains results of all instances with same number of items I, *i.e.*, $|I| \in \{50, 70, 100\}$. Each sub-figure is a scatter plot with one point per solver, whose coordinates are the execution time on y-axis, while the x-axis contains the relative difference between the value z_s of the QUBO yielded by a solver s and the minimum value of the QUBO among all solvers, *i.e.*, $|(z_s - \min_{s' \in \text{solvers}}\{z'_s\}) / \min_{s \in \text{solvers}}\{z'_s\}|$. The closer a point is to the origin of the plane, the better. Fig. S2.1a, S2.1b and S2.1c also show the magnification of the region of the plane containing points closer to the origin.

Differences in results emerge only when considering different number of items: therefore, we present results averaging over all density values and all the 10 instances for each combination of density and number of items, *i.e.*, the coordinates of each point are the average over 40 instances. Values of coordinates of all points of in the figure are reported in Table S2.2.

For QUBO-CARD formulation, where the equality constraint (CARD) is relaxed as QUBO, Gurobi dominates D-Wave for instances with $|I| = 50$ and $|I| = 70$ in both execution time and quality of solution. For instances with higher size, *i.e.*, $|I| = 100$, Gurobi is able to prove optimality of its solution in reasonable time (~ 1.6 seconds), while D-Wave is not able to find good heuristic solutions (~ 8 times higher then optimal).

Table S2.1: Value of coordinates of points in Figure 2 of manuscript

formulation	coordinates	Gurobi 1sec.	D-Wave	random	Gurobi	SA
BINARY	time (sec.)	1	0.274	0.25	55.39	1.53
	$\lvert\frac{(z_s - \min_{s' \in solvers} z'_s)}{\min_{s' \in solvers} z'_s}\rvert$	0.035	5002.79	3.73	0.0001	0.11
UNARY	time (sec.)	1	0.28	0.25	59.54	12.71
	$\lvert\frac{(z_s - \min_{s' \in solvers} z'_s)}{\min_{s' \in solvers} z'_s}\rvert$	0.096	810.47	3.36	0.002	0.15
QUBOCARD	time (sec.)	0.077	0.25	0.25	0.5765	0.92
	$\lvert\frac{(z_s - \min_{s' \in solvers} z'_s)}{\min_{s' \in solvers} z'_s}\rvert$	0.0015	4.33	0.09	0.0013	0.0013
LINEAR	time (sec.)	–	0.24	0.25	0.055	0.79
	$\lvert\frac{(z_s - \min_{s' \in solvers} z'_s)}{\min_{s' \in solvers} z'_s}\rvert$	–	0.055	0.391	2.87E-16	3.02E-16

[1] more information at `https://docs.dwavesys.com/docs/latest/c_qpu_timing.html#qpu-sapi-qpu-timing`

$\|I\|$	coordinate	Gurobi	SA	D-Wave	random	Gurobi 1sec.
50	time (sec.)	47.93	0.88	0.24	0.25	1.00
	$\lvert\frac{(z_s - \min_{s' \in solvers} z'_s)}{\min_{s' \in solvers} z'_s}\rvert$	0.00	0.09	6146.30	1.62	0.03
70	time (sec.)	58.24	1.38	0.27	0.25	1.00
	$\lvert\frac{(z_s - \min_{s' \in solvers} z'_s)}{\min_{s' \in solvers} z'_s}\rvert$	0.00	0.10	4100.36	0.81	0.02
100	time (sec.)	60.00	2.34	0.32	0.25	1.00
	$\lvert\frac{(z_s - \min_{s' \in solvers} z'_s)}{\min_{s' \in solvers} z'_s}\rvert$	0.00	0.14	4761.73	8.79	0.05

(a) BINARY

$\|I\|$	coordinate	Gurobi	SA	D-Wave	random	Gurobi 1sec.
50	time (sec.)	58.62	8.47	0.28	0.25	1.00
	$\lvert\frac{(z_s - \min_{s' \in solvers} z'_s)}{\min_{s' \in solvers} z'_s}\rvert$	0.00	0.15	1409.64	1.86	0.10
70	time (sec.)	60.00	12.99	0.30	0.25	1.00
	$\lvert\frac{(z_s - \min_{s' \in solvers} z'_s)}{\min_{s' \in solvers} z'_s}\rvert$	0.00	0.14	177.24	0.62	0.09
100	time (sec.)	60.00	16.68	0.29	0.25	1.00
	$\lvert\frac{(z_s - \min_{s' \in solvers} z'_s)}{\min_{s' \in solvers} z'_s}\rvert$	0.00	0.17	193.71	7.60	0.10

(b) UNARY

$\|I\|$	coordinate	Gurobi	SA	D-Wave	random	Gurobi 1sec.
50	time(sec.)	0.03	0.45	0.25	0.25	0.03
	$\lvert\frac{(z_s - \min_{s' \in solvers} z'_s)}{\min_{s' \in solvers} z'_s}\rvert$	0	0	0.82	0.06	0.00
70	time(sec.)	0.09	0.77	0.25	0.25	0.03
	$\lvert\frac{(z_s - \min_{s' \in solvers} z'_s)}{\min_{s' \in solvers} z'_s}\rvert$	0	0	4.5	0.06	0.00
100	time(sec.)	1.6	1.54	0.25	0.25	0.12
	$\lvert\frac{(z_s - \min_{s' \in solvers} z'_s)}{\min_{s' \in solvers} z'_s}\rvert$	0	0	7.68	0.11	0.00

(c) QUBO-CARD

$\|I\|$	coordinate	Gurobi	SA	D-Wave	random
50	time (sec.)	0.02	0.47	0.25	0.25
	$\lvert\frac{(z_s - \min_{s' \in solvers} z'_s)}{\min_{s' \in solvers} z'_s}\rvert$	0	0	0.01	0.29
70	time (sec.)	0.02	0.74	0.24	0.25
	$\lvert\frac{(z_s - \min_{s' \in solvers} z'_s)}{\min_{s' \in solvers} z'_s}\rvert$	0	0	0.04	0.39
100	time (sec.)	0.12	1.18	0.25	0.25
	$\lvert\frac{(z_s - \min_{s' \in solvers} z'_s)}{\min_{s' \in solvers} z'_s}\rvert$	0	0	0.11	0.49

(d) LINEAR

Table S2.2: Value of coordinates of points of Figure S2.1

-

Table S2.3: Computational results - BINARY scenario

			Gurobi			SA		D-Wave		random		Gurobi 1sec.	
density	nodes	index	obj. value	time (sec.)	opt. gap	obj. value	time (sec.)	obj. value	time (sec.)	obj. value	time (sec.)	obj. value	time (sec.)
25	50	1	-3205.86	60.00	34465.91%	-2753.56	1.05	7783.58	0.28	23869.58	0.25	-3024.56	1.00
		2	-675.49	60.00	3888.08%	-546.49	0.85	511139.20	0.23	-287.28	0.25	-597.11	1.00
		3	-332.27	45.66	0.00%	-332.27	0.85	94523.42	0.25	-90.00	0.25	-266.57	1.00
		4	-1062.26	60.00	1995.26%	-1033.63	0.77	163045.39	0.23	-216.87	0.25	-1047.52	1.00
		5	-2531.82	60.00	43261.55%	-2206.82	0.98	58531.00	0.28	-419.00	0.25	-2347.82	1.00
		6	-1130.08	6.94	0.00%	-1130.08	0.82	439035.05	0.20	-822.04	0.25	-1130.08	1.00
		7	-597.05	60.00	2932.64%	-579.34	0.89	351527.86	0.26	-169.82	0.25	-579.34	1.00
		8	-3496.00	60.00	51581.37%	-2862.12	0.88	-243.95	0.22	62553.24	0.25	-3268.88	1.01
		9	-1578.11	60.00	8729.99%	-1391.11	0.90	50750.68	0.20	-639.57	0.25	-1493.24	1.00
		10	-1710.66	60.00	18134.03%	-1428.25	0.92	157093.00	0.27	-700.64	0.25	-1571.25	1.00
	70	1	-2735.76	60.00	15159.86%	-2315.62	1.35	97831.67	0.26	-1663.16	0.25	-2673.59	1.00
		2	-585.83	60.00	39943.00%	-554.92	1.40	89069.45	0.29	-108.00	0.25	-577.83	1.00
		3	-1776.34	60.00	12599.30%	-1629.17	1.28	101095.16	0.21	-1170.17	0.25	-1776.34	1.00
		4	-2077.78	60.00	29842.80%	-1560.28	1.45	74713.19	0.30	-723.03	0.25	-1969.50	1.00
		5	-2024.30	60.00	39138.55%	-1742.50	1.50	39402.75	0.21	-593.70	0.25	-2024.30	1.00
		6	-3328.82	60.00	13415.11%	-3277.78	1.36	135564.50	0.27	-2836.00	0.25	-3328.82	1.00
		7	-2090.82	60.00	8114.80%	-2127.28	1.06	30105.98	0.27	9460.02	0.25	-2090.82	1.00
		8	-346.95	60.00	19636.52%	-321.95	1.29	231395.84	0.27	-159.00	0.25	-314.99	1.00
		9	-1564.15	60.00	22192.40%	-1485.15	1.33	69753.60	0.26	-869.00	0.25	-1564.15	1.00
		10	-1976.75	60.01	57439.35%	-1928.55	1.45	-584.47	0.28	-974.17	0.25	-1976.75	1.00
	100	1	-6387.75	60.00	17793.89%	-5027.01	2.58	897314.84	0.33	-3974.00	0.25	-6326.50	1.00
		2	-1609.99	60.00	25589.04%	-1476.24	2.10	2723096.16	0.31	-987.25	0.25	-1457.25	1.00
		3	-3153.32	60.00	69855.92%	-2854.30	2.50	91317.78	0.32	-1384.83	0.25	-2852.47	1.00
		4	-1762.52	60.00	56411.77%	-1386.52	2.42	1733277.06	0.33	-668.00	0.25	-1705.52	1.00
		5	-1582.45	60.00	70220.12%	-1327.29	2.57	1770682.67	0.33	-494.00	0.25	-1582.45	1.00
		6	-2286.29	60.00	56635.29%	-1931.62	2.46	1509581.35	0.33	-506.13	0.25	-2213.41	1.00
		7	-642.66	60.00	93689.38%	-436.66	2.26	4256441.83	0.32	-208.85	0.25	-575.66	1.00
		8	-7484.61	60.00	38607.80%	-6200.50	2.56	918891.01	0.33	-4863.04	0.25	-7416.61	1.00
		9	-258.00	60.00	721942.03%	-93.00	2.18	24402792.04	0.31	-102.00	0.25	-202.00	1.00
		10	-625.20	60.00	182355.35%	-466.71	2.25	5305783.57	0.32	-146.00	0.25	-484.30	1.00

Computational results - BINARY scenario (cont.)

			Gurobi			SA		D-Wave		random		Gurobi 1sec.	
density	nodes	index	obj. value	time (sec.)	opt. gap	obj. value	time (sec.)	obj. value	time (sec.)	obj. value	time (sec.)	obj. value	time (sec.)
50	50	1	-2637.89	60.00	16160.73%	-2228.14	0.90	254717.46	0.27	-798.89	0.25	-2513.29	1.00
		2	-5988.93	60.00	48578.40%	-5060.62	1.05	-1998.30	0.22	48733.30	0.25	-5656.62	1.00
		3	-381.00	19.79	0.00%	-305.00	0.87	17571059.00	0.23	-299.00	0.25	-381.00	1.00
		4	-797.49	50.20	0.00%	-668.49	0.85	2247906.29	0.26	-496.00	0.25	-784.49	1.00
		5	-1510.56	60.00	81549.53%	-1271.02	1.01	255164.74	0.27	2173.97	0.25	-1471.80	1.00
		6	-1073.00	15.52	0.00%	-1058.00	0.81	216722.22	0.20	-917.00	0.25	-1054.00	1.00
		7	-2204.74	60.00	47558.34%	-1948.27	0.94	299209.68	0.27	-1019.00	0.25	-2089.27	1.00
		8	-2684.37	60.00	1391.87%	-2419.00	0.82	204606.42	0.20	-2041.37	0.25	-2684.37	1.00
		9	-2297.42	60.00	19287.87%	-2056.49	0.93	299371.58	0.26	-949.28	0.25	-1951.42	1.00
		10	-1566.31	4.49	0.01%	-1566.31	0.72	292609.03	0.24	-1207.08	0.25	-1566.31	1.00
	70	1	-2147.94	60.00	23549.36%	-1817.94	1.32	1312812.06	0.26	-906.05	0.25	-2147.94	1.00
		2	-6997.50	60.00	93136.18%	-5880.91	1.60	325511.85	0.29	-2889.57	0.25	-6486.48	1.00
		3	-782.62	6.96	0.00%	-655.89	1.19	931367.78	0.27	-189.00	0.25	-782.62	1.00
		4	-677.41	42.62	0.00%	-618.41	1.23	346865.95	0.26	-168.00	0.25	-675.54	1.00
		5	-2951.44	60.00	13260.67%	-2620.00	1.29	1193670.78	0.30	-1008.85	0.25	-2951.44	1.00
		6	-992.88	60.00	13724.23%	-919.47	1.28	325394.95	0.26	-442.87	0.25	-962.88	1.00
		7	-6062.40	60.00	33830.02%	-5094.54	1.51	990359.73	0.28	-3172.00	0.25	-5884.40	1.00
		8	-5330.01	60.00	18286.01%	-4685.01	1.41	1095001.34	0.26	-3197.00	0.25	-5277.01	1.00
		9	-7755.17	60.01	70236.17%	-6755.82	1.53	388476.88	0.29	23866.27	0.25	-7593.52	1.00
		10	-6593.55	60.00	94785.22%	-5467.86	1.64	-4287.86	0.26	-2276.00	0.25	-6402.86	1.00
	100	1	-4091.94	60.00	91113.18%	-3260.94	2.56	4106915.97	0.32	-1648.87	0.25	-3470.94	1.00
		2	-3672.67	60.00	24739.85%	-3131.76	2.08	6337014.38	0.31	-2234.67	0.25	-3672.67	1.00
		3	-10647.35	60.00	24610.72%	-9241.08	2.48	1469411.15	0.32	-7283.00	0.25	-10647.35	1.00
		4	-7403.08	60.00	15276.81%	-6129.38	2.17	722555.92	0.29	-2734.77	0.25	-7403.08	1.00
		5	-1061.02	60.00	257958.24%	-924.02	2.27	8686503.98	0.32	-512.00	0.25	-919.51	1.00
		6	-3550.62	60.00	33345.70%	-3089.00	2.06	5537368.07	0.33	2499.52	0.25	-3550.62	1.00
		7	-4335.00	60.00	14482.61%	-3689.29	1.95	3755610.57	0.33	55396.33	0.25	-4335.00	1.00
		8	-12881.72	60.00	25815.00%	-11503.92	2.60	2325919.06	0.32	-8924.22	0.25	-12131.98	1.00
		9	-3560.00	60.00	27693.33%	-3451.34	2.08	4761820.63	0.30	36120.40	0.25	-3560.00	1.00
		10	-9940.58	60.00	17701.49%	-8625.82	2.40	5306110.17	0.32	-7442.54	0.25	-9734.58	1.00

Computational results - BINARY scenario (cont.)

density	nodes	index	Gurobi obj. value	Gurobi time (sec.)	Gurobi opt. gap	SA obj. value	SA time (sec.)	D-Wave obj. value	D-Wave time (sec.)	random obj. value	random time (sec.)	Gurobi 1sec. obj. value	Gurobi 1sec. time (sec.)
75	50	1	-3543.34	60.00	8177.16%	-3381.34	0.89	816144.31	0.26	-2273.40	0.25	-3543.34	1.00
		2	-271.00	7.46	0.00%	-256.00	0.83	50832980.72	0.23	-262.00	0.25	-271.00	1.00
		3	-3744.53	60.00	4359.47%	-3563.42	0.87	338391.37	0.20	-2895.88	0.25	-3744.53	1.00
		4	-1722.44	60.00	755.12%	-1550.94	0.81	576074.74	0.23	-909.00	0.25	-1722.44	1.00
		5	-4471.66	60.00	5120.25%	-4351.66	0.88	48.29	0.21	-3304.07	0.25	-4471.66	1.00
		6	-726.58	1.97	0.00%	-680.53	0.83	1092998.97	0.23	-250.00	0.25	-726.58	1.00
		7	-758.62	7.93	0.00%	-667.97	0.85	134274.57	0.22	-208.35	0.25	-758.62	1.00
		8	-6280.97	60.00	23671.62%	-5778.76	1.00	159905.85	0.27	-3208.86	0.25	-6226.15	1.00
		9	-1221.70	60.00	15995.26%	-1071.62	0.89	501936.13	0.26	-387.00	0.25	-1021.70	1.00
		10	-1742.16	60.00	1091.28%	-1692.16	0.82	568217.20	0.26	-383.31	0.25	-1627.06	1.00
	70	1	-2000.00	60.00	4517.50%	-1905.00	1.12	2219955.44	0.30	-0.89	0.25	-2000.00	1.00
		2	-5127.78	60.00	47331.20%	-4350.24	1.47	1560795.51	0.28	-1876.00	0.25	-4878.24	1.00
		3	-1421.13	60.00	19114.35%	-1281.13	1.34	932692.15	0.27	-606.00	0.25	-1407.01	1.00
		4	-2675.43	60.00	850.97%	-2596.43	1.29	4582276.70	0.21	-1995.00	0.25	-2675.43	1.00
		5	-4799.77	60.00	66075.70%	-3955.96	1.45	1527700.10	0.28	-2450.83	0.25	-4333.77	1.00
		6	-325.92	60.00	38730.97%	-292.92	1.33	6233613.75	0.27	-204.00	0.25	-312.92	1.00
		7	-979.44	60.00	12027.11%	-817.76	1.31	5534779.05	0.27	-569.00	0.25	-848.44	1.00
		8	-2576.75	60.00	30937.25%	-2324.03	1.41	2047361.11	0.26	-1234.00	0.25	-2493.03	1.00
		9	-2114.81	60.00	7235.30%	-1952.09	1.27	1493169.51	0.27	-1131.38	0.25	-2084.43	1.00
		10	-2397.80	60.00	24728.71%	-2157.42	1.30	2233889.39	0.26	-1054.22	0.25	-2397.80	1.00
	100	1	-2494.28	60.00	38129.16%	-2417.28	2.18	7286292.61	0.31	-1349.00	0.25	-2483.93	1.00
		2	-9853.34	60.01	378958.23%	-8636.04	3.03	2912118.38	0.32	2903031.61	0.25	-8217.69	1.00
		3	-1892.61	60.00	8688.99%	-1866.95	2.10	5550126.14	0.33	-1086.00	0.25	-1892.61	1.00
		4	-2225.65	60.00	257256.00%	-1985.65	2.33	23300313.07	0.32	-1735.00	0.25	-2029.65	1.00
		5	-5902.42	60.00	102737.89%	-5420.16	2.59	5417991.83	0.32	-3121.53	0.25	-5675.42	1.00
		6	-16904.22	60.00	52389.02%	-15188.22	2.73	5194578.94	0.32	-11743.05	0.25	-16835.76	1.00
		7	-7263.94	60.00	38573.92%	-6534.94	2.34	5779231.18	0.32	-4732.64	0.25	-7223.94	1.00
		8	-4707.51	60.01	220065.86%	-4109.85	2.59	7314206.98	0.32	-2582.37	0.25	-4409.22	1.00
		9	-2321.72	60.00	221126.66%	-1908.72	2.39	8799708.04	0.32	-1011.93	0.25	-1985.72	1.00
		10	-15257.81	60.00	16408.49%	-12975.85	2.45	4223911.22	0.32	-10097.20	0.25	-14538.43	1.00

Computational results - BINARY scenario (cont.)

			Gurobi			SA		D-Wave		random		Gurobi 1sec.	
density	nodes	index	obj. value	time (sec.)	opt. gap	obj. value	time (sec.)	obj. value	time (sec.)	obj. value	time (sec.)	obj. value	time (sec.)
100	50	1	-1162.86	32.79	0.00%	-1098.36	0.82	789146.85	0.23	-509.00	0.25	-1162.86	1.00
		2	-6798.69	60.00	30031.18%	-6123.57	0.96	113496.14	0.27	-2910.61	0.25	-6593.69	1.00
		3	-2400.20	60.00	6033.92%	-2255.20	0.85	511107.78	0.26	-612.00	0.25	-2400.20	1.00
		4	-4431.85	60.00	9866.18%	-3979.85	0.83	224650.52	0.23	-3647.85	0.25	-4431.85	1.00
		5	-1566.76	51.42	0.00%	-1565.76	0.86	812074.84	0.23	-967.92	0.25	-1566.76	1.00
		6	-4827.68	60.00	14850.63%	-4604.68	0.86	330169.31	0.20	-3415.70	0.25	-4827.68	1.00
		7	-2819.62	60.00	12794.06%	-2634.62	0.87	888701.84	0.26	-1335.00	0.25	-2819.62	1.00
		8	-876.11	1.89	0.00%	-813.11	0.80	761152.12	0.23	-233.00	0.25	-876.11	1.00
		9	-3957.40	60.00	34964.89%	-3349.40	0.92	495758.39	0.27	-1523.46	0.25	-3738.40	1.00
		10	-1685.32	51.02	0.00%	-1602.16	0.87	47452.74	0.23	-217.36	0.25	-1685.32	1.00
	70	1	-991.25	60.00	2451.70%	-888.25	1.22	2079421.66	0.27	-232.00	0.25	-991.25	1.00
		2	-3429.44	60.00	2449.55%	-3392.49	1.28	445087.95	0.24	-2339.61	0.25	-3429.44	1.00
		3	-3650.00	60.00	96039.39%	-3253.00	1.45	4539431.00	0.28	-2319.00	0.25	-3442.00	1.00
		4	-2032.87	60.00	2268.73%	-1851.87	1.33	4165883.46	0.21	-1211.00	0.25	-1945.87	1.00
		5	-9317.46	60.00	17009.26%	-8266.23	1.38	2508706.58	0.26	-5728.00	0.25	-9317.46	1.00
		6	-6527.60	60.00	39967.31%	-6379.60	1.49	3416409.16	0.26	-4646.79	0.25	-6527.60	1.00
		7	-5864.00	60.00	60988.34%	-5473.00	1.45	4077754.00	0.28	-3562.00	0.25	-5733.00	1.00
		8	-271.00	60.00	155902.45%	-219.00	1.29	32809764.60	0.27	-207.00	0.25	-268.00	1.00
		9	-13411.52	60.00	120820.89%	-11910.76	1.65	129504.78	0.29	-8069.99	0.25	-13074.38	1.00
		10	-10517.94	60.00	154700.85%	-9774.49	1.74	-4904.82	0.27	2802.20	0.25	-10258.49	1.00
	100	1	-14427.46	60.00	11471.98%	-12521.73	2.02	7638388.48	0.33	482240.88	0.25	-13622.36	1.00
		2	-18807.67	60.00	90797.17%	-16315.67	2.74	2560054.45	0.32	-11565.20	0.25	-18176.67	1.00
		3	-8264.64	60.00	47746.50%	-7517.94	2.50	786434.55	0.28	-5105.31	0.25	-8124.64	1.00
		4	-5436.21	60.00	58601.52%	-4644.62	2.34	6822090.82	0.32	-2498.00	0.25	-5119.21	1.00
		5	-1238.42	60.00	41344.57%	-1081.01	2.06	4459618.40	0.31	-273.00	0.25	-1238.42	1.00
		6	-6050.11	60.00	25964.19%	-5651.00	2.06	11898655.40	0.33	103165.56	0.25	-6050.11	1.00
		7	-9438.00	60.00	15919.74%	-8323.00	2.14	8035556.00	0.31	-5899.00	0.25	-9438.00	1.00
		8	-7091.37	60.00	21575.48%	-6416.46	2.20	7335017.07	0.32	-4415.00	0.25	-7091.37	1.00
		9	-1780.00	60.00	112397.66%	-1656.00	2.20	11082851.00	0.32	-960.00	0.25	-1714.00	1.00
		10	-488.11	60.00	176978.08%	-417.11	2.07	9959398.51	0.31	-250.00	0.25	-488.11	1.00

Table S2.4: Computational results - UNARY scenario

			Gurobi			SA		D-Wave		random		Gurobi 1sec.	
density	nodes	index	obj. value	time (sec.)	opt. gap	obj. value	time (sec.)	obj. value	time (sec.)	obj. value	time (sec.)	obj. value	time (sec.)
25	50	1	-3245.56	60.01	53432.34%	-2253.72	30.03	-	-	17605.46	0.25	-2655.94	1.00
		2	-652.11	60	36447.94%	-541.49	5.68	-	-	-275.00	0.25	-541.49	1.00
		3	-266.57	60.01	93623.44%	-332.27	3.8	81631.08	0.29	-90.00	0.25	-191.57	1.00
		4	-1062.26	60	3123.71%	-1055.26	1.1	200.17	0.28	-747.63	0.25	-1052.63	1.00
		5	-2760.8	60.01	106846.58%	-1959.7	26.48	-	-	30825.41	0.25	-2559.82	1.01
		6	-1130.08	60	50.01%	-1130.08	1.76	49833.72	0.27	-830.00	0.25	-1130.08	1.00
		7	-611.34	60	45290.09%	-455.34	4.56	14169.27	0.33	-174.00	0.25	-454.82	1.00
		8	-3267.12	60.01	81154.62%	-2785.12	33.7	-	-	67234.17	0.25	-2436.68	1.01
		9	-1564.81	60	58295.52%	-1093.28	9.72	-	-	-706.13	0.25	-1324.24	1.00
		10	-1710.66	60	74156.20%	-1123.64	15.46	-	-	-749.41	0.25	-1389.69	1.00
	70	1	-2790.59	60	46655.89%	-2025.65	11.37	-	-	-1664.16	0.25	-2195.46	1.00
		2	-602.83	60	258568.69%	-585.83	6.83	-	-	-308.00	0.25	-578.83	1.00
		3	-1790.17	60	25773.59%	-1543	2.69	671.54	0.29	-1182.69	0.25	-1790.17	1.00
		4	-2107.13	60.01	84746.41%	-1477.5	13.28	-	-	-724.03	0.25	-1444.28	1.00
		5	-2096.2	60	94327.47%	-1592.8	14.54	-	-	-778.00	0.25	-2007.50	1.00
		6	-3210.82	60	31878.15%	-3045.96	3.83	26648	0.3	-2517.82	0.25	-3068.96	1.00
		7	-2090.82	60	8191.83%	-1989.91	1.05	75517.3	0.24	8594.16	0.25	-2090.82	1.00
		8	-329.95	60	95417.23%	-300.95	4.2	189152.82	0.29	-159.00	0.25	-225.99	1.00
		9	-1622.05	60	84208.73%	-1276.15	8.51	-	-	-896.15	0.25	-1541.05	1.00
		10	-1999.55	60.01	206265.38%	-1706.69	20.69	-	-	-628.75	0.25	-1652.17	1.00
	100	1	-6395.01	60.01	39715.99%	-4431	19.51	-	-	-3907.00	0.25	-5494.75	1.00
		2	-1598.99	60	53847.66%	-1428.25	4.45	-	-	-1154.00	0.25	-1548.99	1.00
		3	-3253.47	60.01	185911.47%	-2540.32	24.58	-	-	-1763.00	0.25	-2700.32	1.00
		4	-1740.24	60	143692.94%	-1410.81	17.4	-	-	-692.09	0.25	-1345.44	1.00
		5	-1986.45	60	130374.32%	-1359.13	16.23	-	-	-456.00	0.25	-1418.45	1.00
		6	-2354.62	60.01	124605.40%	-1644.21	27.28	-	-	-327.51	0.25	-1954.29	1.01
		7	-514.66	60	352484.25%	-485.4	8	-	-	-161.85	0.25	-514.66	1.00
		8	-7721.61	60.01	54986.04%	-5400.34	30.32	-	-	-4625.04	0.25	-6814.61	1.01
		9	-258	60	4435331.45%	-197	5.77	-	-	-153.00	0.25	-148.00	1.00
		10	-827.2	60	505178.67%	-640.3	12.91	-	-	-229.00	0.25	-539.30	1.00

Computational results - UNARY scenario (cont.)

			Gurobi			SA		D-Wave		random		Gurobi 1sec.	
density	nodes	index	obj. value	time (sec.)	opt. gap	obj. value	time (sec.)	obj. value	time (sec.)	obj. value	time (sec.)	obj. value	time (sec.)
50	50	1	-1593.8	60	154432.32%	-1412.8	13.46	-	-	-584.89	0.25	-2355.89	1.00
		2	-1073	60	1069.22%	-1025	1.57	-	-	23920.00	0.25	-4453.65	1.00
		3	-2180.74	60	163759.62%	-1883.27	13.27	817674.05	0.32	-235.00	0.25	-349.00	1.00
		4	-2684.37	60	4632.20%	-2364.59	1.92	361229.01	0.28	-351.00	0.25	-639.49	1.00
		5	-2162.42	60	121004.09%	-1672.49	8.9	-	-	16279.59	0.25	-1403.80	1.00
		6	-1566.31	4.5	0.01%	-1566.31	0.72	123.57	0.24	-798.82	0.25	-1073.00	1.00
		7	-2118.35	60.01	160364.77%	-1693.35	9.97	-	-	-697.83	0.25	-1845.27	1.00
		8	-6313.91	60.01	105722.18%	-5009.24	37.4	3798.53	0.26	-1993.00	0.25	-2621.37	1.00
		9	-782.62	60	10436.41%	-648.1	3.62	-	-	-1005.00	0.25	-2075.49	1.00
		10	-674.41	60	31387.33%	-585.54	4.21	-77.24	0.19	-1294.00	0.25	-1566.31	1.00
	70	1	-2118.35	60.01	160364.77%	-1693.35	9.97	-	-	-872.05	0.25	-1625.86	1.02
		2	-6313.91	60.01	105722.18%	-5009.24	37.4	-	-	-2791.28	0.25	-5719.24	1.01
		3	-782.62	60	10436.41%	-648.1	3.62	78602.77	0.32	-189.00	0.25	-648.10	1.00
		4	-674.41	60	31387.33%	-585.54	4.21	240287.55	0.3	-226.00	0.25	-613.54	1.00
		5	-3007.22	60	20127.57%	-2549.22	1.78	104963.82	0.3	-2534.87	0.25	-3007.22	1.00
		6	-1026.88	60	48836.59%	-822.81	4.98	-	-	-330.87	0.25	-1026.88	1.00
		7	-6175.54	60.01	90717.15%	-4781.04	23.47	-	-	-3364.68	0.25	-5745.04	1.00
		8	-5243.01	60	64510.52%	-4223	9.37	-	-	-3121.00	0.25	-4710.01	1.00
		9	-7753.18	60.01	81185.06%	-5890.3	47.63	-	-	1345.56	0.25	-6444.82	1.01
		10	-7164.37	60.01	96063.14%	-5179.85	44.99	-	-	-2585.85	0.25	-4874.51	1.01
	100	1	-3797.84	60	626942.92%	-2960.46	29.78	-	-	-1773.72	0.25	-3209.94	1.00
		2	-3675.67	60	52807.96%	-3287.67	5.41	-	-	-2591.00	0.25	-3400.42	1.00
		3	-10868.94	60.01	60281.63%	-8308.34	23.15	-	-	-7190.59	0.25	-9322.49	1.00
		4	-7412.15	60	27578.51%	-5768	5.3	-	-	-208.23	0.25	-7340.15	1.00
		5	-1267.02	60	754065.37%	-995.02	13.98	-	-	-394.51	0.25	-1150.51	1.00
		6	-3550.62	60	39056.35%	-3148	3.11	1406327.46	0.29	-2586.50	0.25	-3550.62	1.00
		7	-4335	60	18351.78%	-3572.29	2.47	89441.37	0.3	52721.03	0.25	-4335.00	1.00
		8	-13019.5	60.01	53704.67%	-10329.88	19.31	-	-	-8683.88	0.25	-12161.52	1.00
		9	-3558	60	27720.75%	-3382.34	2.31	782764.21	0.3	1366.49	0.25	-3558.00	1.00
		10	-9904.58	60	54387.53%	-8150.14	12.58	-	-	-6926.54	0.25	-9245.82	1.00

Computational results - UNARY scenario (cont.)

			Gurobi			SA		D-Wave		random		Gurobi 1sec.	
density	nodes	index	obj. value	time (sec.)	opt. gap	obj. value	time (sec.)	obj. value	time (sec.)	obj. value	time (sec.)	obj. value	time (sec.)
75	50	1	-3666.34	60.01	36453.44%	-3259.57	5.31	-	-	-2371.40	0.25	-3467.91	1.00
		2	-271	60	1675097.66%	-244	3.53	7116469	0.28	-192.00	0.25	-263.00	1.00
		3	-3744.53	60	8735.68%	-3633.53	1.96	483.73	0.29	-2885.88	0.25	-3744.53	1.00
		4	-1722.44	60	16346.90%	-1344.94	3.84	16968.83	0.27	-921.00	0.25	-1722.44	1.00
		5	-4471.66	60	25998.99%	-3722.29	3.65	-367.84	0.31	-3100.00	0.25	-4471.66	1.00
		6	-726.58	60	7664.72%	-599.58	2.74	35884.81	0.27	-250.00	0.25	-588.58	1.00
		7	-758.62	60	21998.10%	-697.16	3.17	11926.72	0.31	-206.35	0.25	-697.16	1.00
		8	-6345.83	60	66613.14%	-5410.51	11.65	-	-	-3254.86	0.25	-5699.76	1.00
		9	-1183.2	60	54827.88%	-1086.42	5.39	-	-	-262.82	0.25	-1043.20	1.00
		10	-1742.16	60	23451.47%	-1341.75	5.72	-	-	-380.31	0.25	-1333.75	1.00
	70	1	-2018	60	3172.08%	-2018	1.38	265592.88	0.24	-979.68	0.25	-2018.00	1.00
		2	-5201.24	60.01	108313.09%	-3991.56	25.09	-	-	-1815.10	0.25	-4875.78	1.00
		3	-1469.01	60	62385.03%	-1310.13	4.76	-	-	-641.00	0.25	-1469.01	1.00
		4	-2675.43	60	3034.35%	-2675.43	2.07	282773.48	0.31	-1969.00	0.25	-2675.43	1.00
		5	-4563.96	60.01	141789.58%	-3802.31	26.29	-	-	-1658.33	0.25	-4278.96	1.00
		6	-301.92	60	305203.70%	-306.92	4.48	179869.38	0.33	-204.00	0.25	-291.92	1.00
		7	-957.44	60	53495.57%	-836.44	4.23	352348.08	0.28	-579.00	0.25	-744.44	1.00
		8	-2618.03	60	128886.86%	-2158.54	10.03	-	-	-1089.00	0.25	-2302.54	1.00
		9	-2052.09	60	40271.53%	-1802.43	4.18	21418.38	0.31	-1116.38	0.25	-2018.52	1.00
		10	-2432.8	60	96733.55%	-1975.47	8.22	-	-	-1002.00	0.25	-2080.42	1.00
	100	1	-2534.28	60	82995.68%	-2286.28	5.51	-	-	-1295.64	0.25	-2375.78	1.00
		2	-9658.35	60.02	578081.75%	-9254.35	97.41	-	-	2360677.56	0.25	-9113.35	1.02
		3	-1892.61	60	19486.03%	-1728.65	3.65	-	-	-1089.66	0.25	-1892.61	1.00
		4	-2439.65	60	792651.48%	-2106.65	11.68	-	-	-1581.00	0.25	-2147.65	1.00
		5	-6285.16	60.01	185492.27%	-5190.16	28.52	-	-	-3258.53	0.25	-5487.16	1.00
		6	-16838.76	60.01	102237.60%	-14707.44	29.59	-	-	-11579.05	0.25	-15386.76	1.01
		7	-7329.95	60	86034.22%	-6233.94	14.68	-	-	-4757.64	0.25	-6547.94	1.00
		8	-4935.85	60.01	307098.52%	-3982.29	33.08	-	-	15939.71	0.25	-4204.85	1.01
		9	-2219.72	60.01	668562.50%	-1905.72	24.14	-	-	-58.37	0.25	-1979.72	1.00
		10	-15174.66	60.01	36579.19%	-11120.65	17.12	-	-	-10136.91	0.25	-14426.78	1.00

Computational results - UNARY scenario (cont.)

			Gurobi			SA		D-Wave		random		Gurobi 1sec.	
density	nodes	index	obj. value	time (sec.)	opt. gap	obj. value	time (sec.)	obj. value	time (sec.)	obj. value	time (sec.)	obj. value	time (sec.)
100	50	1	-1162.86	60	32338.83%	-953.36	3.84	63304.66	0.29	-509.00	0.25	-1131.36	1.00
		2	-6806.69	60	105453.80%	-5684.45	20.24	-	-	-2715.61	0.25	-6583.69	1.00
		3	-2400.2	60.01	30576.22%	-1892.1	6.39	-	-	-628.00	0.25	-2387.00	1.00
		4	-4501.85	60	19926.49%	-3874	2.24	-1276.35	0.3	-3785.00	0.25	-4432.85	1.00
		5	-1566.76	60	21983.89%	-1485.3	2.69	99822.19	0.28	-892.92	0.25	-1485.76	1.00
		6	-4827.68	60	17581.04%	-3844	1.48	-1285.17	0.27	-3660.70	0.25	-4729.68	1.00
		7	-2833.29	60.16	58869.72%	-2399.39	6.46	-	-	-1353.00	0.25	-2360.39	1.00
		8	-876.11	60	4720.46%	-732.11	3.08	6631.76	0.27	-236.00	0.25	-813.11	1.00
		9	-3891.4	60	150479.82%	-3084.29	14.29	-	-	-1550.82	0.25	-3297.39	1.01
		10	-1685.32	60	8791.13%	-1518.32	3.56	5474.51	0.32	-227.00	0.25	-1685.32	1.00
	70	1	-991.25	60	19553.14%	-794.2	3.96	116397.19	0.33	-227.00	0.25	-991.25	1.00
		2	-3398.49	60	26467.11%	-3276.49	3.36	197286.81	0.32	-2403.00	0.25	-3343.09	1.00
		3	-3764	60	268181.19%	-3346	14.65	-	-	-2241.00	0.25	-3391.00	1.00
		4	-2032.87	60	10491.43%	-1737.97	2.62	318453.82	0.32	-1401.00	0.25	-1931.87	1.00
		5	-9317.46	60	64367.11%	-7823.19	11.4	-	-	-5775.45	0.25	-8966.17	1.00
		6	-6715.6	60	131693.66%	-6114.09	9.85	-	-	-4537.79	0.25	-6238.09	1.00
		7	-5782	60	203330.98%	-5147	18.37	-	-	-3388.00	0.25	-5403.00	1.00
		8	-256	60	4099024.46%	-263	4.56	-	-	-186.00	0.25	-230.00	1.00
		9	-13528.52	60.01	117511.75%	-11806.76	39.95	-	-	-7237.99	0.25	-12576.38	1.01
		10	-10570.49	60.01	175355.56%	-9354.49	45.61	-	-	-3400.95	0.25	-9945.49	1.01
	100	1	-14553	60	11703.48%	-12035.82	2.12	1927658.27	0.28	144204.15	0.25	-14427.46	1.00
		2	-18492.67	60.01	89397.85%	-14545.95	39.61	-	-	-11455.00	0.25	-15792.17	1.01
		3	-8206	60	111205.63%	-6997.89	16.06	-	-	-5233.31	0.25	-7605.64	1.00
		4	-5227.21	60	157617.04%	-4565.62	20.84	-	-	-2553.14	0.25	-4888.21	1.00
		5	-1238.42	60	110618.09%	-928.01	5.85	-	-	-222.00	0.25	-1238.42	1.00
		6	-6050.11	60	29839.07%	-5778.05	2.29	1176108.06	0.31	67992.97	0.25	-6050.11	1.00
		7	-9480	60	30091.96%	-8156	5.14	-	-	-7090.00	0.25	-9480.00	1.00
		8	-7091.37	60	70719.03%	-6094.77	7.57	-	-	-4411.00	0.25	-6897.37	1.00
		9	-1928	60	347723.27%	-1626	13.19	-	-	-1027.00	0.25	-1817.00	1.00
		10	-481.11	60	661543.99%	-471.11	5.48	-	-	-226.00	0.25	-412.53	1.00

Table S2.5: Computational results - QUBO-CARD scenario

			Gurobi		SA		D-Wave		random		Gurobi 1sec	
density	nodes	index	obj. value	time (sec.)	obj. value	time (sec.)	obj. value	time (sec.)	obj. value	time (sec.)	obj. value	time (sec.)
25	50	1	-2689.8	0.3	-2689.8	0.49	-2470.23	0.27	-2609.56	0.25	-2689.80	0.31
		2	-318.61	0	-318.62	0.41	-92.55	0.3	-318.62	0.25	-318.62	0.01
		3	-145.43	0	-145.43	0.51	323.75	0.27	-145.43	0.25	-145.43	0.00
		4	-1498.18	0.06	-1498.19	0.51	-1198.84	0.25	-1409.10	0.25	-1498.19	0.06
		5	-1131.89	0.07	-1131.89	0.45	-964	0.25	-1343.50	0.25	-1201.22	0.05
		6	-1748.66	0.02	-1748.66	0.38	-1688.73	0.25	-1748.66	0.25	-1748.66	0.02
		7	-151.19	0	-151.18	0.41	55.8	0.25	-151.18	0.25	-151.18	0.00
		8	-3409.59	0.11	-3409.57	0.42	-3011.64	0.25	-3198.77	0.25	-3409.57	0.08
		9	-712.72	0.17	-712.72	0.43	-568.99	0.25	-657.30	0.25	-712.72	0.14
		10	-677.71	0.11	-677.71	0.45	-317.4	0.25	-415.80	0.25	-627.14	0.12
	70	1	-2414.24	0.07	-2414.24	0.72	-1539.51	0.28	-2298.59	0.25	-2414.24	0.07
		2	-286.26	0.01	-286.26	0.66	1467.1	0.21	-286.26	0.25	-286.26	0.01
		3	-2230.95	0.06	-2230.96	0.74	-1723.09	0.28	-1994.50	0.25	-2230.96	0.05
		4	-813.44	0.04	-813.44	0.61	575.38	0.28	-537.97	0.25	-813.44	0.04
		5	-1469.6	0.45	-1469.6	0.73	-340.1	0.21	-1353.80	0.25	-1469.60	0.38
		6	-6628.47	0.01	-6628.47	0.64	-5082.58	0.28	-5931.40	0.25	-6628.47	0.01
		7	-4254.19	0.06	-4254.19	0.66	-3301.28	0.21	-3632.37	0.25	-4254.19	0.04
		8	-117.98	0	-117.99	0.52	2697.22	0.28	-117.99	0.25	-117.99	0.00
		9	-1280.18	0.08	-1280.19	0.68	314.71	0.21	-1188.52	0.25	-1280.19	0.09
		10	-2814.34	0.13	-2814.34	0.7	-1064.44	0.28	-2722.02	0.25	-2814.34	0.11
	100	1	-11006.83	60	-11006.87	1.44	-6537.03	0.25	-8832.15	0.25	-11006.87	1.00
		2	-2803.37	0.08	-2803.37	1.27	2339.35	0.25	-2479.11	0.25	-2803.37	0.08
		3	-7165.44	0.03	-7165.44	1.21	2475.1	0.25	-6476.93	0.25	-7165.44	0.03
		4	-970.47	0.04	-970.46	1.13	3572.5	0.25	-834.19	0.25	-970.46	0.04
		5	-926.94	0.46	-926.93	1.22	4106.73	0.25	-737.64	0.25	-926.94	0.40
		6	-1427.31	0.09	-1427.31	1.25	2273.7	0.25	-1160.05	0.25	-1427.31	0.08
		7	-298	0	-298	1.06	7570.4	0.25	-291.15	0.25	-298.00	0.00
		8	-15026.48	0.41	-15026.49	1.19	-10037.43	0.25	-12475.74	0.25	-15026.49	0.41
		9	-619.4	0	-619.37	1.74	82442.91	0.25	-619.37	0.25	-619.37	0.00
		10	-698.2	0.01	-698.2	1.19	9579.74	0.25	-697.29	0.25	-698.20	0.01

Computational results - QUBO-CARD scenario (cont.)

density	nodes	index	Gurobi obj. value	Gurobi time (sec.)	SA obj. value	SA time (sec.)	D-Wave obj. value	D-Wave time (sec.)	random obj. value	random time (sec.)	Gurobi 1sec obj. value	Gurobi 1sec time (sec.)
50	50	1	-1499.85	0	-1499.86	0.4	-1417.08	0.25	-1499.86	0.25	-1499.86	0.00
		2	-4557.4	0.04	-4557.39	0.43	-4065.35	0.25	-4013.04	0.25	-4557.39	0.03
		3	-9313.3	0	-9313.34	0.69	17709.68	0.25	-9313.34	0.25	-9313.34	0.00
		4	-1046.62	0	-1046.62	0.59	-293.49	0.25	-1046.62	0.25	-1046.62	0.00
		5	-2116.12	0.02	-2116.13	0.59	-849.22	0.25	-2116.13	0.25	-2116.13	0.01
		6	-953.07	0.04	-953.07	0.48	-463.69	0.25	-735.93	0.25	-953.07	0.04
		7	-1785.75	0.01	-1785.75	0.41	-1385.49	0.25	-1785.75	0.25	-1785.75	0.01
		8	-3401.43	0.07	-3401.43	0.43	-3311.03	0.25	-3171.57	0.25	-3401.43	0.08
		9	-1600.92	0.01	-1600.92	0.43	-1409.68	0.25	-1593.75	0.25	-1600.92	0.01
		10	-2331.16	0.08	-2331.17	0.41	-2109.78	0.25	-1889.85	0.25	-2331.17	0.06
	70	1	-928.78	0	-928.78	0.6	521.73	0.28	-651.95	0.25	-928.78	0.00
		2	-6703.98	0.2	-6704.02	0.65	-5121.93	0.21	-5586.99	0.25	-6704.02	0.16
		3	-48.42	0	-48.42	0.66	725.29	0.28	-27.95	0.25	-48.42	0.00
		4	-120.84	0	-120.84	0.62	1508.74	0.21	-120.84	0.25	-120.84	0.00
		5	-5163.87	0.02	-5163.87	0.74	-3445.36	0.28	-4132.01	0.25	-5163.87	0.03
		6	-431.06	0	-431.06	0.64	1332.46	0.21	-431.06	0.25	-431.06	0.00
		7	-7569.02	0.58	-7568.99	0.72	-6196.06	0.28	-6547.12	0.25	-7568.99	0.56
		8	-7657.91	0.03	-7657.94	0.51	-5813.98	0.21	-6806.94	0.25	-7657.94	0.03
		9	-7640.63	0.01	-7640.66	0.72	-6297.11	0.21	-7016.33	0.25	-7640.66	0.01
		10	-6642.9	0.11	-6642.93	0.68	-5553.49	0.28	-5711.78	0.25	-6642.93	0.08
	100	1	-3711.27	0.45	-3711.27	1.18	4828.63	0.25	-3049.29	0.25	-3711.27	0.39
		2	-6922.75	0.53	-6922.79	1.27	4769.88	0.25	-5327.84	0.25	-6922.79	0.45
		3	-21255.7	0.05	-21255.68	1.22	-11218.51	0.25	-18772.54	0.25	-21255.68	0.04
		4	-16552.97	0.05	-16553	1.2	-8353.46	0.25	-13485.38	0.25	-16553.00	0.04
		5	-1861.43	0.02	-1861.4	1.73	18399.61	0.25	-1861.40	0.25	-1861.40	0.02
		6	-8650.38	0.02	-8650.4	1.14	-2912.24	0.25	-6973.39	0.25	-8650.40	0.02
		7	-8616.73	0.24	-8616.74	1.28	-2666.83	0.25	-6712.44	0.25	-8616.74	0.24
		8	-32518.02	0.04	-32517.97	1.85	-24055.5	0.25	-28131.62	0.25	-32517.97	0.03
		9	-11769.99	0.05	-11770.03	1.6	-1378.66	0.25	-10313.54	0.25	-11770.03	0.04
		10	-31493.99	0.04	-31493.95	1.87	-22646.88	0.25	-28253.67	0.25	-31493.95	0.04

Computational results - QUBO-CARD scenario (cont.)

density	nodes	index	Gurobi obj. value	Gurobi time (sec.)	SA obj. value	SA time (sec.)	D-Wave obj. value	D-Wave time (sec.)	random obj. value	random time (sec.)	Gurobi 1sec obj. value	Gurobi 1sec time (sec.)
75	50	1	-4558.78	0	-4558.78	0.49	-4001.9	0.25	-4518.78	0.25	-4558.78	0.00
		2	-6789.56	0	-6789.48	0.65	73222.28	0.25	-6789.48	0.25	-6789.48	0.00
		3	-4930.82	0.03	-4930.83	0.41	-4706.45	0.25	-4712.13	0.25	-4930.83	0.04
		4	-777.06	0	-777.06	0.41	-447.95	0.25	-777.06	0.25	-777.06	0.00
		5	-5212.88	0	-5212.88	0.38	-5158.69	0.25	-4904.54	0.25	-5212.88	0.00
		6	-100.86	0	-100.86	0.34	95.42	0.25	-100.86	0.25	-100.86	0.00
		7	-194.35	0	-194.35	0.37	618.47	0.25	-194.35	0.25	-194.35	0.00
		8	-7254.44	0.07	-7254.46	0.43	-6571.32	0.25	-6746.32	0.25	-7254.46	0.05
		9	-391.18	0	-391.18	0.39	8.9	0.25	-391.18	0.25	-391.18	0.00
		10	-387.82	0	-387.82	0.41	-362.06	0.25	-377.62	0.25	-387.82	0.00
	70	1	-2323.46	0.02	-2323.45	0.69	-610.03	0.21	-1649.00	0.25	-2323.45	0.01
		2	-3090.26	0	-3090.24	0.62	-1283.88	0.28	-2248.41	0.25	-3090.24	0.00
		3	-471.25	0	-471.25	0.62	2321.04	0.21	-466.13	0.25	-471.25	0.00
		4	-5142.8	0.1	-5142.8	1.01	-3204.57	0.28	-5021.02	0.25	-5142.80	0.09
		5	-3653.72	0.03	-3653.72	0.65	-2609.32	0.21	-3521.88	0.25	-3653.72	0.02
		6	-401.15	0	-401.15	1.01	11466.82	0.28	-401.08	0.25	-401.16	0.00
		7	-1127.36	0	-1127.37	1	2555.77	0.21	-1127.37	0.25	-1127.37	0.00
		8	-1574.51	0	-1574.51	0.64	501.41	0.28	-1490.79	0.25	-1574.51	0.00
		9	-1092.48	0.02	-1092.48	0.64	228.82	0.21	-976.62	0.25	-1092.48	0.01
		10	-1461.91	0.01	-1461.92	0.65	42.73	0.28	-1445.32	0.25	-1461.92	0.01
	100	1	-3011.8	0.14	-3011.79	1.46	5213.76	0.25	-2722.72	0.25	-3011.79	0.12
		2	-32065.95	0.01	-32065.91	2	-10720.65	0.25	-29129.91	0.25	-32065.91	0.01
		3	-1551.05	0.04	-1551.05	1.1	3620.56	0.25	-1199.35	0.25	-1551.05	0.03
		4	-14641.67	0.31	-14641.66	1.96	43332.99	0.25	-14052.95	0.25	-14641.66	0.31
		5	-11937.71	0.02	-11937.66	1.85	845.61	0.25	-10736.56	0.25	-11937.66	0.02
		6	-50195.59	0.03	-50195.55	2.04	-33826.43	0.25	-47086.91	0.25	-50195.55	0.03
		7	-12881.19	0.02	-12881.14	1.74	2779.87	0.25	-10996.62	0.25	-12881.14	0.02
		8	-7811.53	0.62	-7811.49	1.78	3716.92	0.25	-7498.96	0.25	-7811.49	0.50
		9	-4942.32	0.05	-4942.3	1.78	20465.9	0.25	-4820.16	0.25	-4942.30	0.05
		10	-24232.21	0.04	-24232.18	1.47	-18744.15	0.25	-20601.44	0.25	-24232.18	0.04

Computational results - QUBO-CARD scenario (cont.)

			Gurobi		SA		D-Wave		random		Gurobi 1sec	
density	nodes	index	obj. value	time (sec.)	obj. value	time (sec.)	obj. value	time (sec.)	obj. value	time (sec.)	obj. value	time (sec.)
100	50	1	-244.72	0	-244.72	0.35	-93.14	0.25	-244.72	0.25	-244.72	0.00
		2	-5673.51	0.03	-5673.49	0.58	-5578.46	0.25	-5627.64	0.25	-5673.49	0.02
		3	-537.16	0	-537.16	0.43	-431.04	0.25	-535.39	0.25	-537.16	0.00
		4	-3229.49	0	-3229.49	0.41	-3014.96	0.25	-2783.05	0.25	-3229.49	0.00
		5	-1023.39	0.01	-1023.39	0.49	-985	0.25	-979.39	0.25	-1023.39	0.01
		6	-5928.97	0.04	-5929.01	0.57	-5463.86	0.25	-5178.12	0.25	-5929.01	0.03
		7	-1464.37	0	-1464.36	0.4	-1366.58	0.25	-1450.94	0.25	-1464.36	0.00
		8	-77	0	-77	0.29	-74.03	0.25	-74.03	0.25	-77.00	0.00
		9	-2644.11	0	-2644.07	0.56	-2329.8	0.25	-2644.06	0.25	-2644.06	0.00
		10	-527.48	0	-527.48	0.42	-527.48	0.25	-246.64	0.25	-527.48	0.00
	70	1	-112	0	-112	0.58	784.28	0.21	-112.00	0.25	-112.00	0.00
		2	-6465.14	0.03	-6465.09	1	-2918.92	0.28	-5814.35	0.25	-6465.09	0.03
		3	-5900	0.04	-5900	1.05	-1966	0.21	-5432.00	0.25	-5900.00	0.04
		4	-2348.56	0.05	-2348.56	1.08	1395.86	0.28	-2107.36	0.25	-2348.56	0.05
		5	-11751.95	0.19	-11751.99	0.96	-10664.63	0.21	-11171.69	0.25	-11751.99	0.20
		6	-16889.81	0.9	-16889.8	1.16	-13292.15	0.28	-16173.16	0.25	-16889.80	0.84
		7	-11659	0.05	-11659	1.06	-6731	0.21	-10500.00	0.25	-11659.00	0.04
		8	-1427.09	0	-1427.11	1.03	72839.21	0.21	-1427.11	0.25	-1427.11	0.00
		9	-23315.69	0.01	-23315.7	1.1	-21300.59	0.28	-20425.44	0.25	-23315.70	0.01
		10	-20098.52	0.44	-20098.51	1.11	-16935.82	0.21	-18757.84	0.25	-20098.51	0.40
	100	1	-43395.15	0.03	-43395.11	2.01	-31082.14	0.25	-38176.61	0.25	-43395.11	0.03
		2	-27172.06	0.02	-27172.03	1.89	-16085.18	0.25	-22250.52	0.25	-27172.03	0.02
		3	-13791.13	0.02	-13791.14	1.9	627.67	0.25	-11440.08	0.25	-13791.14	0.01
		4	-6162.63	0.12	-6162.62	1.7	8485.55	0.25	-5818.49	0.25	-6162.62	0.09
		5	-162.6	0	-162.6	1.04	3302.1	0.25	-162.60	0.25	-162.60	0.00
		6	-17831.75	0.01	-17831.76	1.88	-2219.1	0.25	-15387.07	0.25	-17831.76	0.01
		7	-18460	0.02	-18460	1.83	-9816	0.25	-15539.00	0.25	-18460.00	0.02
		8	-12030.96	0.01	-12030.92	1.76	115.33	0.25	-10485.47	0.25	-12030.92	0.01
		9	-1627	0.02	-1627	1.67	11298	0.25	-1585.00	0.25	-1627.00	0.02
		10	-423.95	0	-423.94	1.57	16884.83	0.25	-423.94	0.25	-423.94	0.00

Table S2.6: Computational results - LINEAR scenario

			Gurobi		SA		D-Wave		random	
density	nodes	index	obj. value	time (sec.)	obj. value	time (sec.)	obj. value	time (sec.)	obj. value	time (sec.)
25	50	1	-5413.34	0	-5413.34	0.42	-5353.05	0.25	-5101.39	0.25
		2	-1947.99	0	-1947.99	0.37	-1947.99	0.25	-1852.14	0.25
		3	-3381.34	0	-3381.34	0.35	-3381.34	0.25	-3194.18	0.25
		4	-2059.46	0	-2059.46	0.44	-1598.61	0.25	-1000.42	0.25
		5	-5699	0	-5699	0.42	-5617	0.25	-5149.00	0.25
		6	-1237.55	0	-1237.55	0.36	-1237.55	0.25	-1136.08	0.25
		7	-1454.76	0	-1454.76	0.41	-1416.47	0.25	-1387.73	0.25
		8	-7667.74	0	-7667.74	0.43	-7667.74	0.25	-7407.54	0.25
		9	-3036.88	0.02	-3036.88	0.48	-2757.22	0.25	-1712.76	0.25
		10	-2449.4	0.01	-2449.4	0.43	-2193.6	0.25	-2057.00	0.25
	70	1	-4161.06	0.01	-4161.06	0.71	-3828.71	0.21	-3098.43	0.25
		2	-2426.25	0	-2426.25	0.64	-2210.03	0.21	-2339.76	0.25
		3	-5091.56	0.01	-5091.56	0.49	-3572.91	0.28	-1215.31	0.25
		4	-3736.84	0.01	-3736.84	0.64	-2234.13	0.21	-2774.50	0.25
		5	-3907.2	0	-3907.2	0.69	-3243.32	0.21	-3389.09	0.25
		6	-3590.33	0	-3590.33	0.66	-3307	0.28	-3203.61	0.25
		7	-5381.88	0.02	-5381.88	0.62	-4578.76	0.21	-1491.39	0.25
		8	-1938.76	0	-1938.76	0.57	-1938.76	0.21	-1852.67	0.25
		9	-2671.63	0	-2671.63	0.65	-2671.63	0.28	-2255.25	0.25
		10	-6830.61	0	-6830.61	0.58	-6830.61	0.21	-6162.21	0.25
	100	1	-14557.07	0.02	-14557.07	0.96	-11626.17	0.25	-5360.22	0.25
		2	-2173.51	0.02	-2173.51	1.1	981.47	0.25	-1423.54	0.25
		3	-10840.88	0	-10840.88	0.86	-10360.32	0.25	-8718.74	0.25
		4	-8463.77	0.02	-8463.77	1	-4633.78	0.25	-3714.62	0.25
		5	-10043.61	0.01	-10043.61	0.99	-9116.29	0.25	-3910.19	0.25
		6	-14718.59	0.01	-14718.59	0.97	-13486.02	0.25	-4415.39	0.25
		7	-2850.84	0	-2850.84	0.87	-1571.73	0.25	-2744.19	0.25
		8	-11297	0	-11297	0.95	-8370.26	0.25	-7839.62	0.25
		9	-15746.47	0	-15746.47	0.74	-15746.47	0.25	-14678.72	0.25
		10	-6236.01	0	-6236.01	0.83	-6174.61	0.25	-5920.22	0.25

Computational results - LINEAR scenario (cont.)

			Gurobi		SA		D-Wave		random	
density	nodes	index	obj. value	time (sec.)	obj. value	time (sec.)	obj. value	time (sec.)	obj. value	time (sec.)
50	50	1	-4627.5	0.01	-4627.5	0.48	-4627.5	0.25	-4522.19	0.25
		2	-12016.64	0	-12016.64	0.46	-12016.64	0.25	-10918.55	0.25
		3	-79600.17	0	-79600.17	0.35	-79600.17	0.25	-70890.83	0.25
		4	-6584.51	0	-6584.51	0.39	-6584.51	0.25	-5920.87	0.25
		5	-9668.55	0	-9668.55	0.48	-9668.55	0.25	-9337.90	0.25
		6	-12244.42	0.01	-12244.42	0.37	-12244.42	0.25	-1025.32	0.25
		7	-6365.99	0	-6365.99	0.46	-6365.99	0.25	-6126.19	0.25
		8	-3580.59	0	-3580.59	0.47	-3580.59	0.25	-2336.17	0.25
		9	-5069.63	0	-5069.63	0.47	-5069.63	0.25	-4726.03	0.25
		10	-6887.82	0.01	-6887.82	0.48	-6887.82	0.25	-1559.62	0.25
	70	1	-7920.81	0.02	-7920.81	0.75	-6300.41	0.28	-4026.17	0.25
		2	-13442.84	0.01	-13442.84	0.76	-13442.84	0.21	-11471.02	0.25
		3	-17665.15	0.01	-17665.15	0.63	-17665.15	0.21	-1165.86	0.25
		4	-14202.56	0.01	-14202.56	0.64	-14202.56	0.28	-1506.10	0.25
		5	-6283.72	0.01	-6283.72	0.81	-5900.17	0.21	-2157.83	0.25
		6	-7163.45	0.01	-7163.45	0.76	-7163.45	0.21	-2081.08	0.25
		7	-11652.04	0	-11652.04	0.7	-11652.04	0.28	-10253.15	0.25
		8	-8623.89	0	-8623.89	0.76	-8266.89	0.21	-6188.96	0.25
		9	-16633.91	0.02	-16633.91	0.77	-16633.91	0.21	-12141.22	0.25
		10	-13461.43	0.02	-13461.43	0.78	-13461.43	0.28	-11857.51	0.25
	100	1	-20885.83	0.55	-20885.83	1.18	-19704.14	0.25	-9952.21	0.25
		2	-5766.33	0.26	-5766.33	1.16	-4359.75	0.25	-3974.86	0.25
		3	-20394.51	0.31	-20394.51	1.24	-17453.65	0.25	-11651.63	0.25
		4	-12547.64	0.47	-12547.64	1.25	-11921.25	0.25	-5084.62	0.25
		5	-11858.25	0	-11858.25	1.02	-11858.25	0.25	-11064.82	0.25
		6	-5431.28	0.02	-5431.28	1.3	-4508.52	0.25	-2836.37	0.25
		7	-37180.33	0.01	-37180.33	0.98	-37180.33	0.25	-2554.71	0.25
		8	-19818.5	0.01	-19818.5	1.3	-14995.01	0.25	-13040.29	0.25
		9	-4722.11	0.03	-4722.11	1.26	-2839.14	0.25	-1796.43	0.25
		10	-15427.48	0	-15427.48	1.13	-14083.56	0.25	-9930.60	0.25

Computational results - LINEAR scenario (cont.)

density	nodes	index	Gurobi obj. value	Gurobi time (sec.)	SA obj. value	SA time (sec.)	D-Wave obj. value	D-Wave time (sec.)	random obj. value	random time (sec.)
75	50	1	-5934.41	0	-5934.41	0.51	-5934.41	0.25	-5848.87	0.25
		2	-194255.92	0	-194255.92	0.32	-194255.92	0.25	-180949.96	0.25
		3	-4563.94	0	-4563.94	0.54	-4563.94	0.25	-3257.40	0.25
		4	-7310.16	0.01	-7310.16	0.5	-7310.16	0.25	-2566.30	0.25
		5	-5198.56	0	-5198.56	0.58	-5190.44	0.25	-4658.91	0.25
		6	-2394.43	0	-2394.43	0.49	-2394.43	0.25	-2219.01	0.25
		7	-2668.34	0	-2668.34	0.53	-2668.34	0.25	-2576.80	0.25
		8	-9797.91	0.01	-9797.91	0.6	-9522.24	0.25	-9044.03	0.25
		9	-4287.59	0.01	-4287.59	0.57	-4287.59	0.25	-4100.66	0.25
		10	-16159.62	0.01	-16159.62	0.5	-16159.62	0.25	-2272.82	0.25
	70	1	-21909.69	0.01	-21909.69	0.71	-21909.69	0.21	-1280.84	0.25
		2	-23901.85	0.09	-23901.85	0.83	-23901.85	0.21	-9049.95	0.25
		3	-19534.76	0.08	-19534.76	0.75	-19534.76	0.28	-3236.83	0.25
		4	-4094.11	0	-4094.11	0.94	-4094.11	0.28	-2882.66	0.25
		5	-15206.76	0.33	-15206.76	0.78	-15206.76	0.21	-11016.57	0.25
		6	-8972.57	0	-8972.57	0.76	-8972.57	0.21	-8461.19	0.25
		7	-6901.04	0	-6901.04	0.7	-6901.04	0.28	-5937.19	0.25
		8	-18592.66	0.14	-18592.66	0.74	-18592.66	0.28	-5836.12	0.25
		9	-36856.95	0.01	-36856.95	0.61	-36856.95	0.21	-2293.20	0.25
		10	-21854.58	0.01	-21854.58	0.74	-21854.58	0.21	-4415.06	0.25
	100	1	-46611.48	0.01	-46611.48	1.12	-46611.48	0.25	-3574.01	0.25
		2	-55943.48	0	-55943.48	1.42	-55943.48	0.25	-52764.73	0.25
		3	-68551.73	0.01	-68551.73	1.04	-68551.73	0.25	-1956.47	0.25
		4	-30298.94	0	-30298.94	1.11	-30298.94	0.25	-26733.55	0.25
		5	-20443.82	0.87	-20443.82	1.55	-19060.28	0.25	-19096.70	0.25
		6	-30620.36	0	-30620.36	1.46	-29969.18	0.25	-24890.24	0.25
		7	-22573.75	0.41	-22573.75	1.46	-21792.88	0.25	-10334.09	0.25
		8	-21690.61	0	-21690.61	1.41	-21277.81	0.25	-20455.34	0.25
		9	-23704.37	0	-23704.37	1.23	-23704.37	0.25	-21725.42	0.25
		10	-62291.65	0.01	-62291.65	1.22	-62291.65	0.25	-13745.80	0.25

Computational results - LINEAR scenario (cont.)

			Gurobi		SA		D-Wave		random	
density	nodes	index	obj. value	time (sec.)	obj. value	time (sec.)	obj. value	time (sec.)	obj. value	time (sec.)
		1	-10283.72	0.04	-10283.72	0.53	-10283.72	0.25	-2895.20	0.25
		2	-14475.95	0.01	-14475.95	0.58	-14353.08	0.25	-13600.98	0.25
		3	-27423.14	0	-27423.14	0.4	-27423.14	0.25	-3238.05	0.25
		4	-24212.61	0	-24212.61	0.42	-24212.61	0.25	-4258.27	0.25
	50	5	-3489.21	0.01	-3489.21	0.52	-3489.21	0.25	-2906.26	0.25
		6	-7412.01	0.1	-7412.01	0.57	-7412.01	0.25	-4136.59	0.25
		7	-7670.5	0.54	-7670.5	0.6	-7670.5	0.25	-5348.16	0.25
		8	-24122.65	0	-24122.65	0.44	-24122.65	0.25	-1529.79	0.25
		9	-10760.8	0.01	-10760.8	0.81	-10760.8	0.25	-9827.11	0.25
		10	-35767.71	0	-35767.71	0.38	-35767.71	0.25	-1711.46	0.25
		1	-48682.71	0.01	-48682.71	0.67	-48682.71	0.28	-2043.59	0.25
		2	-6033.68	0	-6033.68	0.88	-6033.68	0.21	-4929.67	0.25
		3	-17700	0	-17700	0.86	-17700	0.21	-15937.00	0.25
		4	-5489.86	0	-5489.85	0.89	-5489.86	0.21	-3516.83	0.25
100	70	5	-32465.68	0.01	-32465.68	0.8	-32465.68	0.28	-10076.67	0.25
		6	-16789.24	0	-16789.24	0.94	-16789.24	0.28	-15249.83	0.25
		7	-21600	0	-21600	0.88	-21600	0.21	-19160.00	0.25
		8	-40863.42	0	-40863.42	0.65	-40863.42	0.21	-38204.19	0.25
		9	-35412.97	0	-35412.97	0.86	-35412.97	0.21	-28796.35	0.25
		10	-35142.21	0	-35142.21	0.88	-35142.21	0.21	-30744.21	0.25
		1	-52087.77	0.18	-52087.77	1.29	-52087.77	0.25	-9318.91	0.25
		2	-77629.3	0.01	-77629.3	1.3	-77629.3	0.25	-24426.16	0.25
		3	-26467.59	0.53	-26467.59	1.57	-26467.59	0.25	-14153.92	0.25
		4	-78353.49	0.01	-78353.49	1.18	-78353.49	0.25	-13697.27	0.25
	100	5	-162819.89	0.01	-162819.89	0.84	-162819.89	0.25	-2030.97	0.25
		6	-8079.28	0.79	-8079.28	1.62	-8079.28	0.25	-3006.54	0.25
		7	-67395	0.01	-67395	1.23	-67395	0.25	-7075.00	0.25
		8	-61831.77	0.01	-61831.77	1.25	-61831.76	0.25	-8219.63	0.25
		9	-47027	0.01	-47027	1.3	-47027	0.25	-9944.00	0.25
		10	-29483.61	0.33	-29483.61	1.53	-29483.6	0.25	-4574.60	0.25

Table S2.7: Results KPI $\Pi(S)$

				potential gain		SA								D-Wave							
						BINARY		UNARY		QUBO-CARD		LINEAR		BINARY		UNARY		QUBO-CARD		LINEAR	
density	nodes	index	k	w	$\Pi(S)$	w	$\Pi(S)$	w	$\Pi(S)$	w	$\Pi(S)$	w	$\Pi(S)$	w	$\Pi(S)$	w	$\Pi(S)$	w	$\Pi(S)$	w	$\Pi(S)$
25	50	1	12	7	0.12635	0	1.00000	3	0.43041	0	1.00000	8	0.09109	12	0.02230	-	-	2	0.57388	5	0.23690
		2	4	19	0.13663	15	0.22736	14	0.25578	1	0.92000	21	0.10313	0	1.00000	-	-	18	0.15614	15	0.22736
		3	2	17	0.43102	19	0.37959	20	0.35510	22	0.30857	29	0.17143	18	0.40490	10	0.63673	19	0.37959	3	0.88245
		4	11	14	0.01608	35	0.00000	33	0.00000	32	0.00000	16	0.00766	14	0.01608	35	0.00000	28	0.00002	17	0.00518
		5	9	1	0.82000	3	0.54388	2	0.66939	1	0.82000	2	0.66939	4	0.43973	-	-	7	0.22508	3	0.54388
		6	10	31	0.00001	30	0.00002	30	0.00002	37	0.00000	35	0.00000	17	0.00901	18	0.00628	37	0.00000	38	0.00000
		7	3	31	0.04944	23	0.14923	21	0.18643	8	0.58571	19	0.22934	25	0.11735	20	0.20714	24	0.13265	28	0.07857
		8	12	4	0.32052	7	0.12635	8	0.09109	0	1.00000	0	1.00000	1	0.76000	-	-	7	0.12635	0	1.00000
		9	7	21	0.01563	15	0.06732	10	0.18665	5	0.45432	22	0.01185	16	0.05386	-	-	21	0.01563	29	0.00116
		10	8	8	0.21985	2	0.70286	3	0.58571	10	0.14324	10	0.14324	10	0.14324	-	-	16	0.03382	9	0.17797
	70	1	11	42	0.00001	31	0.00077	31	0.00077	15	0.05530	14	0.06881	34	0.00028	-	-	30	0.00107	37	0.00009
		2	3	18	0.40373	21	0.33657	18	0.40373	18	0.40373	17	0.42795	20	0.35806	-	-	23	0.29622	17	0.42795
		3	12	47	0.00000	51	0.00000	51	0.00000	56	0.00000	8	0.20304	23	0.00491	39	0.00001	34	0.00012	35	0.00008
		4	8	39	0.00084	27	0.01536	27	0.01536	9	0.31194	23	0.03331	22	0.03997	-	-	27	0.01536	31	0.00652
		5	8	31	0.00652	26	0.01877	24	0.02764	4	0.60841	30	0.00815	20	0.05687	-	-	26	0.01877	33	0.00409
		6	16	47	0.00000	41	0.00000	43	0.00000	12	0.03224	32	0.00001	19	0.00289	26	0.00017	44	0.00000	42	0.00000
		7	15	43	0.00000	53	0.00000	54	0.00000	5	0.28743	33	0.00001	32	0.00002	20	0.00312	50	0.00000	13	0.03057
		8	2	38	0.20538	25	0.40994	30	0.32298	11	0.70849	31	0.30683	28	0.35652	30	0.32298	22	0.46708	21	0.48696
		9	8	20	0.05687	13	0.17504	11	0.23489	20	0.05687	19	0.06745	22	0.03997	-	-	29	0.01012	27	0.01536
		10	10	13	0.10885	3	0.62514	3	0.62514	7	0.32217	2	0.73292	3	0.62514	-	-	0	1.00000	3	0.62514
	100	1	23	50	0.00000	37	0.00000	43	0.00000	4	0.34512	13	0.02584	19	0.00384	-	-	15	0.01393	31	0.00005
		2	11	61	0.00001	61	0.00001	61	0.00001	28	0.02134	21	0.06381	25	0.03458	-	-	57	0.00004	49	0.00034
		3	13	25	0.01780	15	0.10390	11	0.19774	10	0.23112	27	0.01213	17	0.07439	-	-	9	0.26964	6	0.42362
		4	7	10	0.46674	13	0.36504	11	0.43044	9	0.50564	1	0.93000	7	0.59182	-	-	6	0.63944	8	0.54728
		5	6	29	0.12014	23	0.19890	24	0.18340	22	0.21547	41	0.03780	1	0.94000	-	-	31	0.10056	66	0.00113
		6	8	1	0.92000	1	0.92000	1	0.92000	0	1.00000	14	0.28514	12	0.34541	-	-	15	0.25861	17	0.21196
		7	3	34	0.28299	29	0.35346	5	0.85600	28	0.36883	41	0.20105	42	0.19082	-	-	36	0.25766	16	0.58926
		8	24	42	0.00000	26	0.00022	33	0.00001	3	0.43476	17	0.00575	2	0.57576	-	-	13	0.02148	17	0.00575
		9	2	82	0.03091	59	0.16566	74	0.06566	13	0.75576	84	0.02424	87	0.01576	-	-	78	0.04667	12	0.77333
		10	4	37	0.15191	31	0.22047	32	0.20769	9	0.68159	36	0.16204	17	0.46863	-	-	62	0.01882	28	0.26236

Results KPI $\Pi(S)$ (cont.)

density	nodes	index	k	potential gain w	potential gain $\Pi(S)$	SA BINARY w	SA BINARY $\Pi(S)$	SA UNARY w	SA UNARY $\Pi(S)$	SA QUBO-CARD w	SA QUBO-CARD $\Pi(S)$	SA LINEAR w	SA LINEAR $\Pi(S)$	D-Wave BINARY w	D-Wave BINARY $\Pi(S)$	D-Wave UNARY w	D-Wave UNARY $\Pi(S)$	D-Wave QUBO-CARD w	D-Wave QUBO-CARD $\Pi(S)$	D-Wave LINEAR w	D-Wave LINEAR $\Pi(S)$
50	50	1	7	17	0.04277	12	0.12635	2	0.73714	2	0.73714	20	0.02038	0	1.00000	-	-	12	0.12635	15	0.06732
		2	12	12	0.02230	1	0.76000	5	0.23690	2	0.57388	5	0.23690	7	0.12635	-	-	4	0.32052	8	0.09109
		3	3	29	0.06786	31	0.04944	33	0.03469	0	1.00000	17	0.27837	16	0.30531	32	0.04163	28	0.07857	0	1.00000
		4	4	34	0.00790	18	0.15614	23	0.07620	13	0.28678	16	0.20137	7	0.53587	27	0.03845	25	0.05493	13	0.28678
		5	6	10	0.24155	6	0.44423	2	0.77224	13	0.14630	16	0.08463	2	0.77224	-	-	2	0.77224	10	0.24155
		6	8	35	0.00001	32	0.00008	35	0.00001	7	0.27010	6	0.33012	14	0.05636	31	0.00014	30	0.00023	21	0.00799
		7	7	5	0.45432	2	0.73714	0	1.00000	5	0.45432	0	1.00000	3	0.62964	-	-	3	0.62964	8	0.27010
		8	11	31	0.00000	27	0.00004	30	0.00000	12	0.03221	19	0.00227	19	0.00227	28	0.00002	29	0.00001	30	0.00000
		9	7	20	0.02038	17	0.04277	15	0.06732	2	0.73714	20	0.02038	3	0.62964	-	-	12	0.12635	9	0.22508
		10	9	40	0.00000	40	0.00000	38	0.00000	38	0.00000	24	0.00125	15	0.02818	27	0.00033	39	0.00000	35	0.00000
	70	1	6	13	0.27676	9	0.42348	6	0.57182	34	0.01486	38	0.00691	2	0.83478	-	-	15	0.22110	23	0.08189
		2	14	13	0.03982	7	0.19346	8	0.15047	8	0.15047	13	0.03982	7	0.19346	-	-	3	0.50639	0	1.00000
		3	2	22	0.46708	3	0.91553	8	0.78302	12	0.68447	9	0.75776	17	0.57060	31	0.30683	29	0.33954	14	0.63768
		4	2	54	0.04969	54	0.04969	52	0.06335	11	0.70849	36	0.23230	46	0.11429	40	0.18012	46	0.11429	63	0.00870
		5	13	49	0.00000	50	0.00000	49	0.00000	37	0.00001	15	0.03057	19	0.01003	42	0.00000	39	0.00000	42	0.00000
		6	4	36	0.05058	40	0.02989	32	0.08051	11	0.49638	32	0.08051	45	0.01380	-	-	40	0.02989	26	0.14806
		7	14	1	0.80000	2	0.63768	6	0.24763	1	0.80000	0	1.00000	5	0.31561	-	-	11	0.06881	0	1.00000
		8	14	31	0.00008	25	0.00086	24	0.00124	2	0.63768	17	0.01244	4	0.40058	-	-	30	0.00012	27	0.00041
		9	15	25	0.00048	10	0.07373	16	0.01200	5	0.28743	5	0.28743	6	0.22110	-	-	11	0.05530	26	0.00032
		10	14	9	0.11649	3	0.50639	3	0.50639	0	1.00000	8	0.15047	0	1.00000	-	-	7	0.19346	0	1.00000
	100	1	10	6	0.52230	12	0.26075	2	0.80909	21	0.08323	7	0.46674	0	1.00000	-	-	3	0.72653	21	0.08323
		2	12	71	0.00000	71	0.00000	69	0.00000	16	0.10757	7	0.39658	7	0.39658	-	-	58	0.00001	65	0.00000
		3	21	43	0.00000	28	0.00038	23	0.00196	12	0.04875	22	0.00268	1	0.79000	-	-	30	0.00019	40	0.00000
		4	19	61	0.00000	53	0.00000	51	0.00000	7	0.21722	56	0.00000	60	0.00000	-	-	50	0.00000	59	0.00000
		5	5	47	0.03812	46	0.04201	47	0.03812	10	0.58375	36	0.10127	25	0.22925	-	-	22	0.28041	5	0.76959
		6	14	81	0.00000	81	0.00000	81	0.00000	67	0.00000	74	0.00000	29	0.00545	71	0.00000	60	0.00000	73	0.00000
		7	17	76	0.00000	77	0.00000	80	0.00000	100	0.00000	2	0.68747	31	0.00090	26	0.00345	52	0.00000	40	0.00006
		8	24	35	0.00000	38	0.00000	41	0.00000	0	1.00000	21	0.00142	34	0.00001	-	-	27	0.00015	32	0.00002
		9	15	80	0.00000	79	0.00000	78	0.00000	100	0.00000	25	0.00900	48	0.00002	39	0.00028	60	0.00000	78	0.00000
		10	23	57	0.00000	60	0.00000	59	0.00000	8	0.11308	50	0.00000	16	0.01016	-	-	42	0.00000	51	0.00000

Results KPI $\Pi(S)$ (cont.)

						SA								D-Wave							
				potential gain		BINARY		UNARY		QUBO-CARD		LINEAR		BINARY		UNARY		QUBO-CARD		LINEAR	
density	nodes	index	k	w	$\Pi(S)$	w	$\Pi(S)$	w	$\Pi(S)$	w	$\Pi(S)$	w	$\Pi(S)$	w	$\Pi(S)$	w	$\Pi(S)$	w	$\Pi(S)$	w	$\Pi(S)$
75	50	1	9	14	0.03758	11	0.08458	13	0.04965	25	0.00082	18	0.01120	2	0.66939	-	-	20	0.00571	19	0.00805
		2	2	36	0.07429	42	0.02286	39	0.04490	12	0.57388	20	0.35510	20	0.35510	21	0.33143	35	0.08571	12	0.57388
		3	11	31	0.00000	29	0.00001	32	0.00000	0	1.00000	18	0.00345	22	0.00057	20	0.00146	29	0.00001	32	0.00000
		4	5	25	0.02508	23	0.03810	23	0.03810	15	0.15322	30	0.00732	18	0.09504	26	0.02006	24	0.03105	26	0.02006
		5	11	20	0.00146	14	0.01608	16	0.00766	10	0.06189	19	0.00227	7	0.15399	1	0.78000	19	0.00227	20	0.00146
		6	2	44	0.01224	47	0.00245	46	0.00490	6	0.77224	43	0.01714	34	0.09796	36	0.07429	44	0.01224	45	0.00816
		7	2	16	0.45796	6	0.77224	14	0.51429	3	0.88245	9	0.66939	13	0.54367	17	0.43102	11	0.60490	16	0.45796
		8	12	10	0.04602	5	0.23690	6	0.17373	12	0.02230	11	0.03221	3	0.43041	-	-	13	0.01526	11	0.03221
		9	3	7	0.62964	11	0.46628	11	0.46628	27	0.09036	13	0.39643	7	0.62964	-	-	3	0.82730	14	0.36429
		10	3	6	0.67571	4	0.77449	1	0.94000	5	0.72398	1	0.94000	5	0.72398	-	-	12	0.43041	22	0.16714
	70	1	9	56	0.00000	56	0.00000	56	0.00000	0	1.00000	4	0.56915	17	0.06814	52	0.00000	57	0.00000	15	0.09777
		2	9	16	0.08177	4	0.56915	10	0.22732	8	0.31194	7	0.36393	4	0.56915	-	-	12	0.16374	19	0.04678
		3	4	17	0.31937	6	0.69296	16	0.34492	39	0.03432	26	0.14806	5	0.73841	-	-	7	0.64965	41	0.02590
		4	9	55	0.00000	55	0.00000	55	0.00000	16	0.08177	45	0.00003	31	0.00326	42	0.00011	56	0.00000	55	0.00000
		5	9	15	0.09777	18	0.05657	8	0.31194	3	0.65747	6	0.42348	2	0.75776	-	-	12	0.16374	20	0.03853
		6	2	50	0.07867	26	0.39172	50	0.07867	18	0.54907	57	0.03230	48	0.09565	67	0.00124	54	0.04969	20	0.50725
		7	4	32	0.08051	36	0.05058	33	0.07203	5	0.73841	11	0.49638	6	0.69296	32	0.08051	39	0.03432	6	0.69296
		8	6	7	0.51821	11	0.34365	5	0.62997	0	1.00000	6	0.57182	4	0.69296	-	-	15	0.22110	13	0.27676
		9	6	40	0.00453	38	0.00691	36	0.01026	12	0.30870	5	0.62997	14	0.24763	23	0.08189	33	0.01773	5	0.62997
		10	6	28	0.04001	24	0.07144	25	0.06212	15	0.22110	30	0.02927	23	0.08189	-	-	30	0.02927	26	0.05384
	100	1	7	71	0.00010	67	0.00027	71	0.00010	20	0.19845	28	0.09203	36	0.03881	-	-	47	0.00963	53	0.00393
		2	17	3	0.56822	6	0.31664	1	0.83000	7	0.25937	7	0.25937	0	1.00000	-	-	17	0.03064	4	0.46863
		3	6	65	0.00136	64	0.00163	67	0.00093	36	0.06290	42	0.03395	59	0.00377	-	-	78	0.00006	35	0.06929
		4	8	38	0.01817	30	0.05073	33	0.03505	32	0.03972	39	0.01582	24	0.10133	-	-	47	0.00476	31	0.04493
		5	12	16	0.10757	4	0.59468	3	0.67864	4	0.59468	17	0.09220	7	0.39658	-	-	6	0.45462	0	1.00000
		6	23	31	0.00005	19	0.00384	23	0.00098	5	0.26243	22	0.00139	11	0.04718	-	-	20	0.00275	11	0.04718
		7	14	28	0.00676	33	0.00221	26	0.01032	0	1.00000	26	0.01032	5	0.46258	-	-	32	0.00279	29	0.00545
		8	10	2	0.80909	6	0.52230	7	0.46674	3	0.72653	7	0.46674	3	0.72653	-	-	31	0.01964	10	0.33048
		9	7	9	0.50564	2	0.86424	15	0.30834	7	0.59182	6	0.63944	10	0.46674	-	-	15	0.30834	5	0.69030
		10	22	50	0.00000	50	0.00000	52	0.00000	7	0.16504	2	0.60667	12	0.04135	-	-	48	0.00000	41	0.00000

Results KPI $\Pi(S)$ (cont.)

				potential gain		SA								D-Wave							
						BINARY		UNARY		QUBO-CARD		LINEAR		BINARY		UNARY		QUBO-CARD		LINEAR	
density	nodes	index	k	w	$\Pi(S)$	w	$\Pi(S)$	w	$\Pi(S)$	w	$\Pi(S)$	w	$\Pi(S)$	w	$\Pi(S)$	w	$\Pi(S)$	w	$\Pi(S)$	w	$\Pi(S)$
100	50	1	3	28	0.07857	25	0.11735	29	0.06786	38	0.01122	20	0.20714	27	0.09036	33	0.03469	23	0.14923	23	0.14923
		2	10	13	0.03391	8	0.14324	13	0.03391	17	0.00901	24	0.00052	9	0.10914	-	-	6	0.24155	20	0.00292
		3	4	17	0.17768	13	0.28678	19	0.13663	22	0.08891	28	0.03176	19	0.13663	-	-	2	0.84490	28	0.03176
		4	11	28	0.00002	19	0.00227	21	0.00093	6	0.20532	4	0.35715	20	0.00146	26	0.00007	24	0.00021	6	0.20532
		5	5	22	0.04639	18	0.09504	15	0.15322	43	0.00001	35	0.00142	16	0.13133	4	0.64696	18	0.09504	29	0.00960
		6	12	34	0.00000	32	0.00000	31	0.00000	10	0.04602	10	0.04602	19	0.00116	11	0.03221	35	0.00000	34	0.00000
		7	6	16	0.08463	8	0.33012	9	0.28296	3	0.67571	3	0.67571	14	0.12257	-	-	9	0.28296	17	0.06970
		8	2	17	0.43102	12	0.57388	22	0.30857	10	0.63673	6	0.77224	20	0.35510	17	0.43102	2	0.92082	5	0.80816
		9	7	18	0.03370	11	0.15399	10	0.18665	1	0.86000	3	0.62964	8	0.27010	-	-	17	0.04277	5	0.45432
		10	2	26	0.22531	24	0.26531	22	0.30857	13	0.54367	18	0.40490	11	0.60490	27	0.20653	26	0.22531	14	0.51429
	70	1	2	41	0.16812	41	0.16812	12	0.68447	5	0.86128	57	0.03230	15	0.61491	46	0.11429	45	0.12422	5	0.86128
		2	9	50	0.00000	48	0.00001	49	0.00000	43	0.00007	42	0.00011	51	0.00000	45	0.00003	51	0.00000	13	0.13834
		3	8	2	0.78302	8	0.35815	9	0.31194	2	0.78302	1	0.88571	13	0.17504	-	-	14	0.15047	4	0.60841
		4	6	44	0.00176	45	0.00135	44	0.00176	22	0.09359	43	0.00226	43	0.00226	41	0.00362	43	0.00226	4	0.69296
		5	14	13	0.03982	5	0.31561	12	0.05249	1	0.80000	15	0.02253	7	0.19346	-	-	16	0.01679	12	0.05249
		6	12	20	0.01141	16	0.03224	17	0.02508	1	0.82857	20	0.01141	8	0.20304	-	-	18	0.01940	2	0.68447
		7	11	21	0.01346	14	0.06881	20	0.01726	4	0.49638	7	0.28458	18	0.02792	-	-	26	0.00354	4	0.49638
		8	2	47	0.10476	48	0.09565	51	0.07081	3	0.91553	37	0.21863	39	0.19255	-	-	53	0.05631	2	0.94327
		9	17	5	0.23710	3	0.42795	9	0.06814	1	0.75714	3	0.42795	0	1.00000	-	-	10	0.04915	5	0.23710
		10	15	7	0.16928	7	0.16928	6	0.22110	5	0.28743	5	0.28743	1	0.78571	-	-	10	0.07373	1	0.78571
	100	1	23	74	0.00000	72	0.00000	73	0.00000	76	0.00000	1	0.77000	50	0.00000	46	0.00000	58	0.00000	37	0.00000
		2	20	3	0.50810	2	0.63838	0	1.00000	1	0.80000	4	0.40334	3	0.50810	-	-	2	0.63838	5	0.31931
		3	13	12	0.16885	9	0.26964	1	0.87000	20	0.04432	22	0.03101	10	0.23112	-	-	10	0.23112	11	0.19774
		4	9	34	0.01946	35	0.01680	34	0.01946	20	0.12191	15	0.21645	31	0.02979	-	-	8	0.45670	30	0.03419
		5	2	52	0.22788	50	0.24747	53	0.21838	19	0.65455	27	0.53091	26	0.54566	-	-	34	0.43333	20	0.63838
		6	14	77	0.00000	77	0.00000	81	0.00000	18	0.04976	5	0.46258	50	0.00002	56	0.00000	53	0.00001	80	0.00000
		7	16	71	0.00000	70	0.00000	71	0.00000	12	0.10757	0	1.00000	10	0.15950	-	-	55	0.00000	12	0.10757
		8	12	55	0.00003	56	0.00002	54	0.00004	0	1.00000	38	0.00206	25	0.02487	-	-	40	0.00133	43	0.00060
		9	5	30	0.16076	12	0.52035	33	0.12828	36	0.10127	35	0.10971	40	0.07254	-	-	29	0.17294	17	0.38000
		10	2	24	0.57576	19	0.65455	42	0.33394	11	0.79111	13	0.75576	1	0.98000	-	-	46	0.28909	33	0.44000

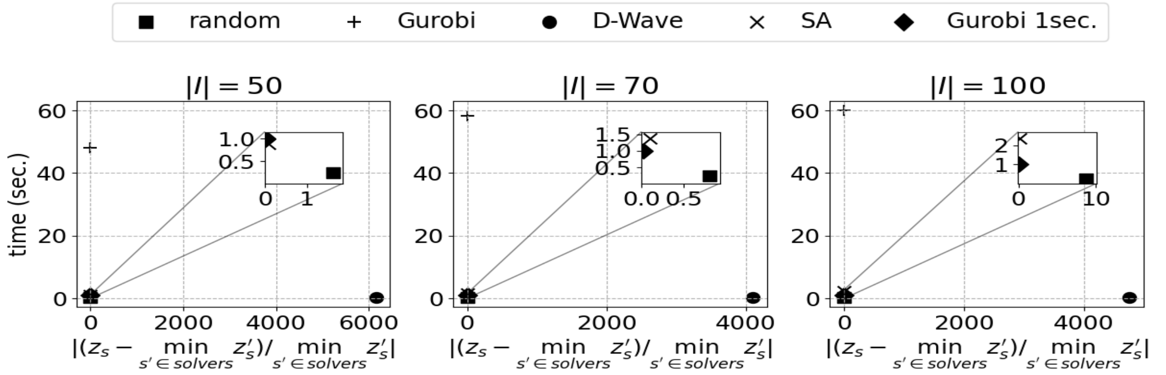

(a) BINARY

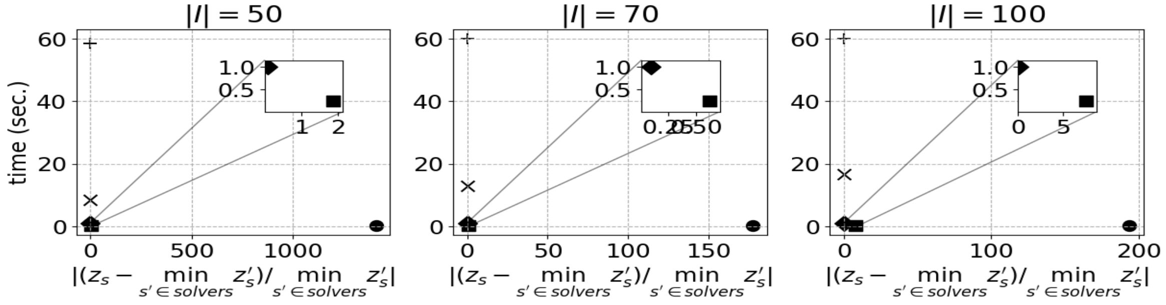

(b) UNARY

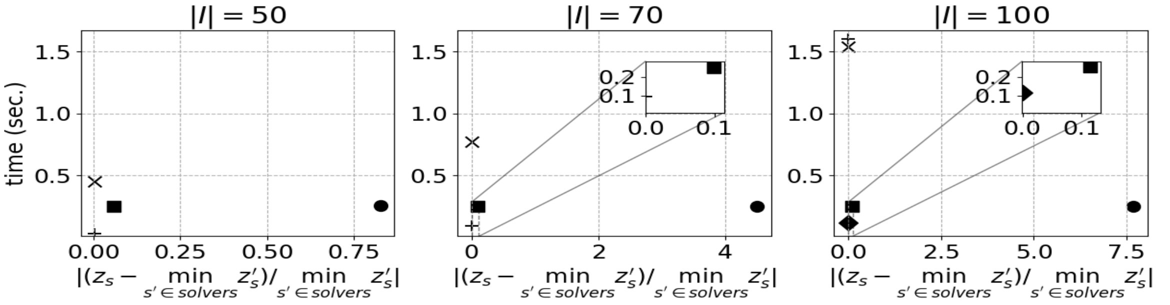

(c) QUBO-CARD

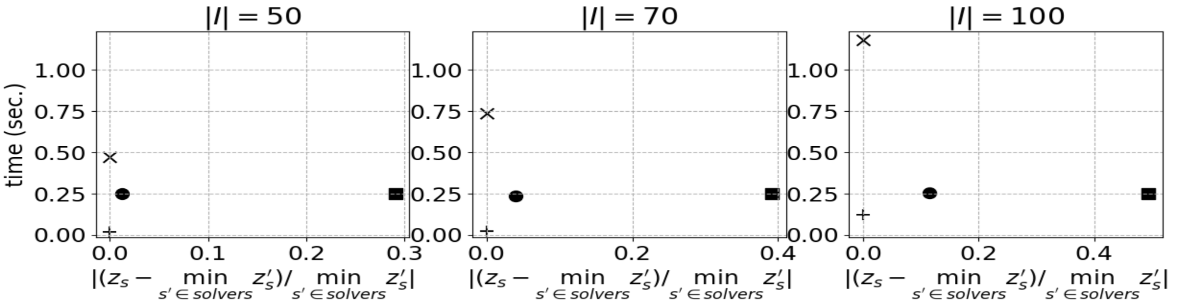

(d) LINEAR

Figure S2.1: Scatter plots of execution time (on y-axis) and the relative difference between the objective function value resulting from a solver and the minimum among all solvers (on x-axis), *i.e.*, $|\frac{z_s - \min_{s' \in \text{solvers}} z_{s'}}{\min_{s' \in \text{solvers}} z_{s'}}|$. Values are averaged on all instances with same number of items.

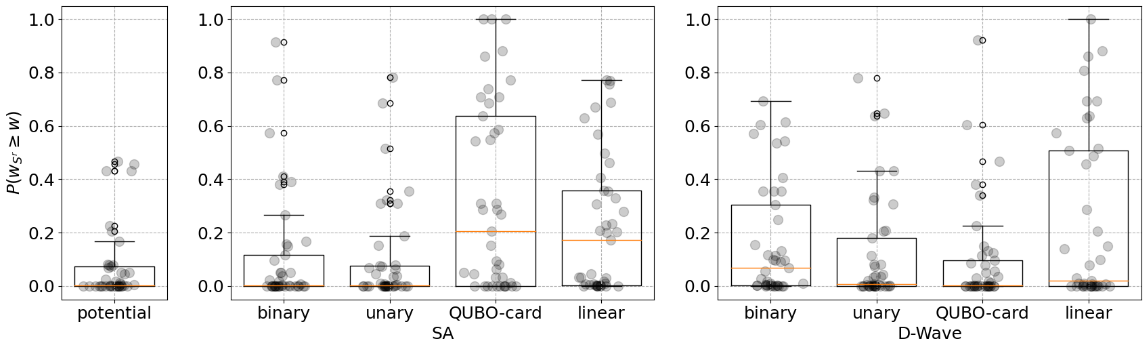

Figure S3.2: Boxplots of KPI $\Pi(S)$ considering only the 41 instances with a solution for the D-Wave UNARY formulation: median values are 0.0018 for the potential gain sorting, 0.002 for SA-BINARY, 0.0017 for SA-UNARY, 0.17 for SA-LINEAR, 0.20 for SA-QUBO-CARD, 0.06 for D-Wave-BINARY, 0.002 for D-Wave-QUBO-CARD, 0.02 for D-Wave-LINEAR and 0.006 for D-Wave-UNARY.

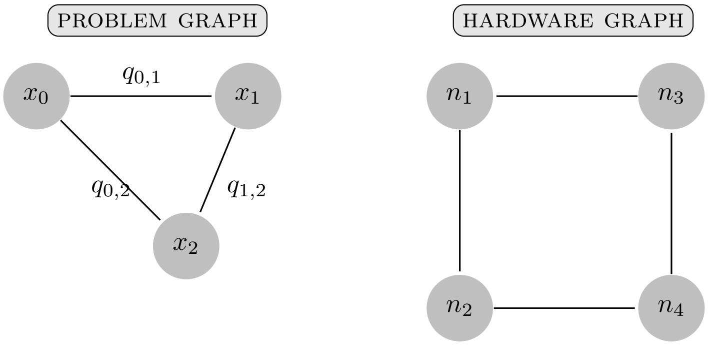

Figure S4.3: Problem interaction graph (left) and hardware graph (right) on which to embed the problem

S3 KPI $\Pi(S)$ for D-Wave unary scenario

Fig. S3.2 shows boxplots of values of KPI $\Pi(S)$ restricting to the 41 instances for which the execution of D-Wave in UNARY formulation was possible. The first block is related to the sorting provided by the naïve measure of potential gain; the second and third block contain results of the sortings computed from the set of solutions created during the execution of SA and D-Wave, respectively.

From this figure we can see that D-Wave with UNARY formulation has worse performance than both the potential gain and D-Wave with QUBO-CARD formulation. The conclusions we can draw from the figure do not differ from those drawn from the discussion of Section 4.1.2 of the paper, where results of all instances were considered.

S4 Example of embedding

We hereby give a simple example of embedding of a problem interaction graph within the graph of an hardware, and the relationship between a 'chain' and 'chain strength' and the mathematical model.

Let us consider the following QUBO, with 3 variables x_0, x_1 and x_2:

$$\min q_{0,1}x_0x_1 + q_{0,2}x_0x_2 + q_{1,2}x_1x_2 \tag{2}$$

The interaction graph of this QUBO is shown in Fig. S4.3, on the left. As hardware, let us consider a graph with 4 nodes whose connections form a square, as shown in Fig. S4.3, on the right.

A direct mapping between the variables of the problem and the graph, and which covers all interactions, is not possible. A transformation of the problem is needed. The usual approach is to make copies of variables;

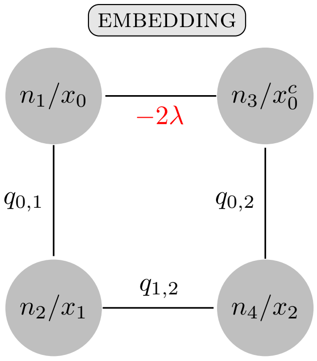

Figure S4.4: Embedding

connections of a variable are split among its copies in disjoint sets, with the additional constraint that copies of the same variable must have same value.

In QUBO (2) of our example, only one variable needs to be duplicated to produce an interaction graph that can be embedded in the hardware graph. Let us duplicate variable x_0 with the new variable x_0^c, and let x_0 be linked to x_1, while its copy x_0^c is connected to x_2. A new constraint forcing $x_0 = x_0^c$ is added to the model. The resulting formulation is the following:

$$\min \; q_{0,1}x_0x_1 + q_{0,2}x_0^c x_2 + q_{1,2}x_1x_2 \tag{3}$$

$$\text{s.t. } x_0 = x_0^c \tag{4}$$

The resulting embedding is shown in Fig. S4.4, where variable x_0 is assigned to nodes n_1 and its copy x_0^c to n_3, x_1 to n_2 and x_2 to n_4. Non-zero quadratic coefficients $q_{i,j}$ are 'weights' of the links in the hardware graph connecting the corresponding variables x_i and x_j.

The additional constraint (4) ensuring same value for the copies must be relaxed to comply with the QUBO format of the function to optimize. We add to the objective function the violation of (4) squared, with the following final formulation:

$$\min \; q_{0,1}x_0x_1 + q_{0,2}x_0^c x_2 + q_{1,2}x_1x_2 + \lambda(x_0 - x_0^c)^2 \tag{5}$$

This new violation term need a penalty multiplier, λ, as any other violation term added to the objective function. Given that $\lambda(x_0 - x_0^c)^2 = \lambda x_0^2 - 2\lambda x_0 x_0^c + \lambda x_0^c$, x_0 and its copy x_0^c have a non-zero quadratic coefficient (-2λ), which is the 'weight' of the link connecting x_0 and x_0^c in the hardware graph. The value of the multiplier λ providing the best relaxation has to be found.

In D-Wave jargon, the set of copies of a variable is called 'chain', and 'chain strength' is the penalty multiplier λ of the violations of the equality constraint between the copies of a variable. In order to get the best relaxation possible it is required to consider the setting of value of 'chain strength' together with the setting of values of all other multipliers. With a general purpose solver, D-Wave proposes a built-in function to set 'chain strength' heuristically, such as 'uniform torque compensation'.
